# Ion drugs for precise orthotopic tumor management by *in situ* the generation of toxic ion and drug pools

**DOI:** 10.7150/thno.66468

**Published:** 2022-01-01

**Authors:** Yushuo Feng, Ruixue Qin, Lihua Xu, Xiaoqian Ma, Dandan Ding, Shi Li, Lei Chen, Yaqing Liu, Wenjing Sun, Hongmin Chen

**Affiliations:** State Key Laboratory of Molecular Vaccinology and Molecular Diagnostics and Center for Molecular Imaging and Translational Medicine, School of Public Health, Xiamen University, Xiamen 361102, China.

**Keywords:** Tumor acidic environment, Internal ions overload, Chemodynamic therapy, Calcification, Anti-inflammatory

## Abstract

**Background:** Asymmetric intracellular and extracellular ionic gradients are critical to the survivability of mammalian cells. Given the importance of manganese (Mn^2+^), calcium (Ca^2+^), and bicarbonate (HCO_3_^-^) ions, any alteration of the ion-content balance could induce a series of cellular responses. HCO_3_^-^ plays an indispensable role for Mn-mediated Fenton-like reaction, but this is difficult to achieve because bicarbonates are tightly regulated by live cells, and are limited in anticancer efficacy.

**Methods:** A responsive and biodegradable biomineral, Mn-doped calcium carbonate integrated with dexamethasone phosphate (DEX) (Mn:CaCO_3_-DEX), was reported to enable synergistic amplification of tumor oxidative stress, reduce inflammation, and induce Ca-overload cell apoptosis by elevating the intracellular and extracellular ionic gradients.

**Results:** Under the acidic environment in tumor region, the ions (Mn^2+^, CO_3_^2-^, Ca^2+^) were released by the degradation of Mn:CaCO_3_-DEX and then escalated oxidative stresses by triggering a HCO_3_^-^-indispensable Mn-based Fenton-like reaction and breaking Ca^2+^ ion homeostasis to cause oxidative stress in cells and calcification. The released anti-inflammatory and antitumor drug, DEX, could alleviate the inflammatory environment. The investigations *in vitro* and *in vivo* demonstrated that the synergistic oncotherapy could effectively inhibit the growth of subcutaneous tumors and orthotopic liver tumors. Notably, normal cells showed greater tolerance of the synergistic influences.

**Conclusion:** As an ion drug, Mn:CaCO_3_-DEX is an excellent potential diagnostic agent for precise orthotopic tumor management by the generation *in situ* of toxic ion and drug pools in the environment of tumor region, with synergistic effects of enhanced chemodynamic therapy, calcification, and anti-inflammation effects.

## Introduction

Tumors are described as “a wound that doesn't heal”, and inflammation is a hallmark of cancer 1-5. In the environment of tumor region, inflammatory cells and secreted inflammatory factors are important components that can release chemicals including reaction oxygen species (ROS). They added some complexity to the tumor environment 6-9. Dexamethasone phosphate (DEX), a glucocorticoid and commercial drug using for inflammatory diseases 10-11, has been increasingly used in malignant tumor treatment in recent years 12-16. It can result in tumor inhibition and tumor environment modulation, also reduce hematopoietic toxicity and immune response of chemotherapeutic agents 17.

Calcium (Ca^2+^) is a ubiquitous but important modulator of cellular physiology and is associated with diverse physiological events 18-22. The intracellular Ca^2+^ overload was recognized as important to increase cell apoptosis factors, and may induce mitochondrial dysfunction 23-28. Therefore, destruction of the intracellular Ca^2+^ homeostasis via calcium overload, is a potential method for antitumor therapy. Calcium channels played a very important role in the homeostasis of intracellular Ca^2+^ ions by restricting the movement of calcium ions into cells 29-30. This is limited to inducing influx of Ca^2+^ into cells and facilitating release of Ca^2+^ due to the protective mechanism of cells via Ca^2+^ excretion 31-32. Calcium carbonate (CaCO_3_) is a calcium-containing nanostructures and excellent pH responsive material; it is a good choice for biological applications 33-37.

Chemodynamic therapy (CDT) which depends on neither O_2_ nor external light sources and could produce highly deleterious ROS via the reaction of metal ions (Fe^2+^, Cu^+^ and Mn^2+^) and H_2_O_2_ 38-44. The generation of toxic hydroxyl radicals (·OH) causes oxidative damages to lipids molecules with many vital biological functions and dysfunction of mitochondrial to activate cell apoptosis 45-51. Mn ions are essential human dietary elements that have been the focus of imaging-guided CDT agents 52-55. In Mn^2+^-mediated CDT, bicarbonates (HCO_3_^-^) play a decisive role, versus Fe^2+^-mediated Fenton reaction. Although the physiological buffers could provide few certain amounts of HCO_3_^-^ to support Mn^2+^-mediated Fenton-like reaction, they are limited to the inhibition of tumor growth because bicarbonates are tightly regulated by live cells 56-58. Thus, the self-supplied *in situ* HCO_3_^-^ and Mn^2+^ pools could overcome the challenge 59.

**Scheme 1:**
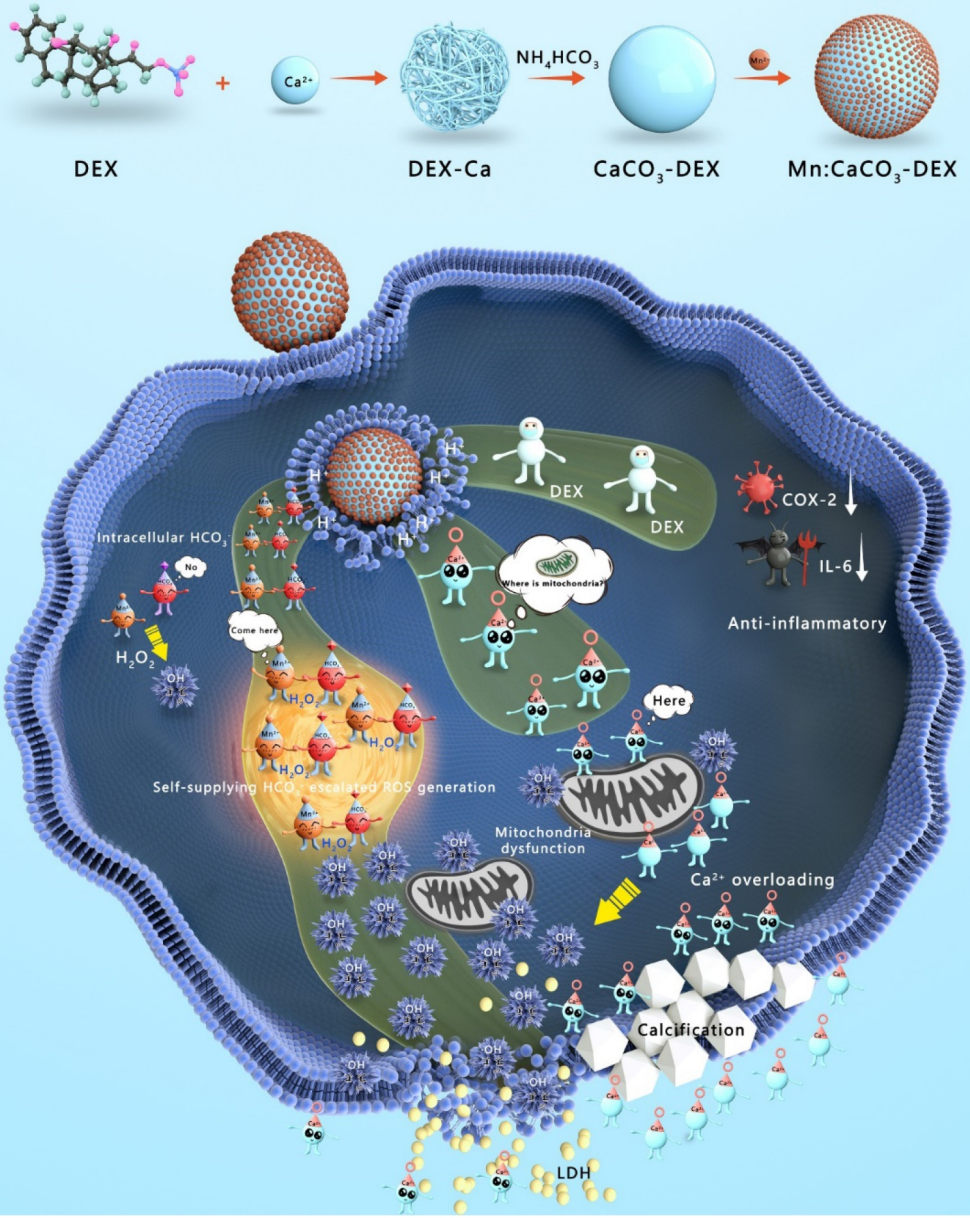
** Synthesis and Functions of Mn:CaCO_3_-DEX in the environment of tumor region.** The Mn:CaCO_3_-DEX reacted with the acidic environment in tumor region and released metal ions (Mn^2+^ and Ca^2+^), HCO_3_^-^, and DEX. The calcium-overload resulted in oxidative stress. The generated HCO_3_^-^ amplified Mn^2+^ Fenton-like peroxidation reactions to generate toxic ·OH, and also regulated the intracellular pH to manage tumor growth. The released DEX drugs relieved inflammation and modulated the tumor environment to provide additional tumor inhibitory effects. The therapeutic profile of Mn:CaCO_3_-DEX on subcutaneous tumor models and orthotopic liver tumor models and showed efficient synergistic tumor therapy.

Herein, Mn:CaCO_3_-DEX biomineral was synthesized according to a gas diffusion procedure to enable synergistic amplification of tumor oxidative stress, reduction of inflammation, and induction of Ca-overload cell apoptosis (Scheme 1). The CaCO_3_ matrix was recognized as an excellent pH-responsive and biodegradable biominerals. The Mn:CaCO_3_-DEX reacted with the acidic environment in tumor region and released metal ions (Mn^2+^ and Ca^2+^), HCO_3_^-^, and DEX. The calcium-overload resulted in oxidative stress. The generated HCO_3_^-^ amplified Mn^2+^-mediated Fenton-like peroxidation reactions to generate toxic ·OH, and also regulated the intracellular pH to manage tumor growth. The released DEX drugs relieved inflammation and modulated the tumor environment to provide additional tumor inhibitory effects^60^. Significantly, the released Mn^2+^ pools from Mn:CaCO_3_-DEX can be used for ultra-sensitive magnetic resonance (MR) imaging to guide this ion drug for precise tumor management. The therapeutic profile of Mn:CaCO_3_-DEX was systematically investigated on subcutaneous tumor models and orthotopic liver tumor models and showed efficient synergistic tumor therapy. These results demonstrated that Mn:CaCO_3_-DEX offers the synergistic effects of enhanced CDT, calcification, and anti-inflammation effects.

## Results and Discussion

Dexamethasone sodium phosphate (DEX) is a synthetic glucocorticoid and commercial drug used for inflammatory diseases and antitumor, containing a phosphate group 61. Metal ions (Ca^2+^, Mn^2+^) coordinated with phosphate groups of DEX, and formed nanofiber network of Ca-DEX with filamentous structures (Figure S1). Gas (CO_2_ and NH_3_) were diffused into the solution of Ca-DEX for the formation of controllable CaCO_3_ particles in an enclosed environment. By simply modulating the feeding ratios of DEX to Ca^2+^, the reaction times in the NH_4_HCO_3_ environment, the morphologies and drug load capacities could be precisely tuned (Figure S2). Finally, we chose a formula to prepare calcium carbonate-dexamethasone sodium phosphate (CaCO_3_-DEX) with a feeding ratio of DEX to CaCl_2_·2H_2_O of 6/50, and a reaction time of 8 h.

**Figure 1:**
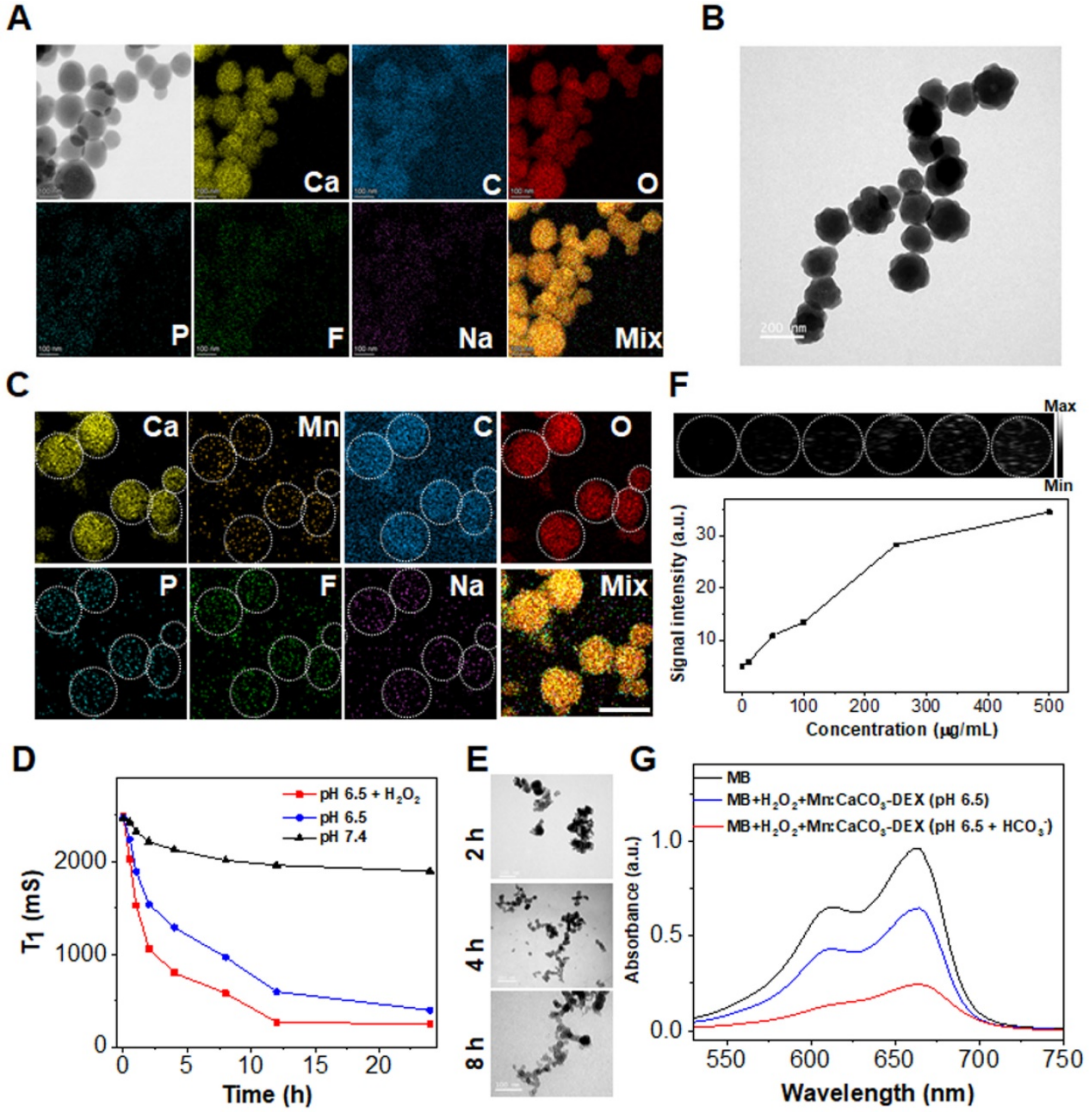
** Preparation and characterization of Mn:CaCO_3_-DEX**. **(A)** Scanning transmission electron microscope (STEM) images and corresponding element mapping of CaCO_3_-DEX (scale bar: 100 nm). **(B)** TEM image of Mn:CaCO_3_-DEX (scale bar: 200 nm). **(C)** STEM images and corresponding element mapping of Mn:CaCO_3_-DEX (scale bar: 100 nm). **(D)**The Mn release behaviors from Mn:CaCO_3_-DEX in different solutions. **(E)** TEM images of Mn:CaCO_3_-DEX after incubation with different time in acidic environment (pH 6.5 + H_2_O_2_) (scale bar: 100 nm). **(F)** Ultrasound images and signal intensity of the generation of CO_2_ in acidic environment (pH 6.5 + H_2_O_2_). **(G)** MB degradation by *in situ* self-suppling HCO_3_^-^-enhanced Mn-mediated Fenton-like reaction.

An average diameter of 150.4 ± 20.2 nm of CaCO_3_-DEX were achieved (Figure 1A). The P, F, and Na atoms in the scanning transmission electron microscopy (STEM), and the typical characterized peak (242 nm) proved the existence of DEX in the CaCO_3_-DEX nanoparticles (Figure 1A; Figure S3). The Mn^2+^ ions were coordinated with free phosphate groups of CaCO_3_-DEX to form Mn:CaCO_3_-DEX (Figure 1B), and there are almost no changes in morphology. The STEM and EDS analyses confirmed that the isolated manganese atoms were anchored on the matrix of CaCO_3_-DEX (Figure 1C; Figure S4). The hydrodynamic size and zeta potential of the nanomaterial proved that PAH was successfully modified to improve water solubility (Figure S5). Mn:CaCO_3_-DEX was dispersed well in water, PBS (pH 7.4), fetal bovine serum (FBS) and cell culture medium (DMEM). It had kept long-term stability (Figure S6). To further verify the stability of Mn^2+^, we also verified the release of Mn^2+^ in DMEM. As shown in Figure S7, the release of Mn^2+^ in DMEM was lower than 15%.

The simulated healthy body internal environment and tumor environment (i.e., neutral (pH=7.4), and acidic environment (pH=6.5 pH=6.5 with 1 mM H_2_O_2_, and pH=4.0) revealed that the Mn^2+^, Ca^2+^, CO_2_ bubbles and DEX drugs were released after stimulation (this work monitored the MR signals of Mn^2+^ (Figure 1D), Ca^2+^ and Mn^2+^ concentrations (ICP-OES, Figure S8A, B), US imaging signals of CO_2_ (Figure 1F), and the absorbance of DEX (Figure S7C). The morphology and structure changes were evaluated by TEM, confirming the TME-stimulative biodegradation and release process (Figure 1E).

The released *in situ* HCO_3_^-^ is indispensable to accelerate the Fenton-like reaction between Mn^2+^ and intracellular H_2_O_2_ to produce hydroxyl radical (·OH) 62. As revealed in Figure 1G, the absorbance of MB decreased dramatically by 33.3% (blue line) after the incubation of Mn:CaCO_3_-DEX plus H_2_O_2_. Notably, the absorbance of MB decreased by 75.0% (red line) in the presence of HCO_3_^-^ (the highest theoretical concentration that was released from the Mn:CaCO_3_ matrix). This proved that the released *in situ* HCO_3_^-^ in the acidic environment of tumor region could escalate an accelerated Fenton-like reaction.

**Figure 2:**
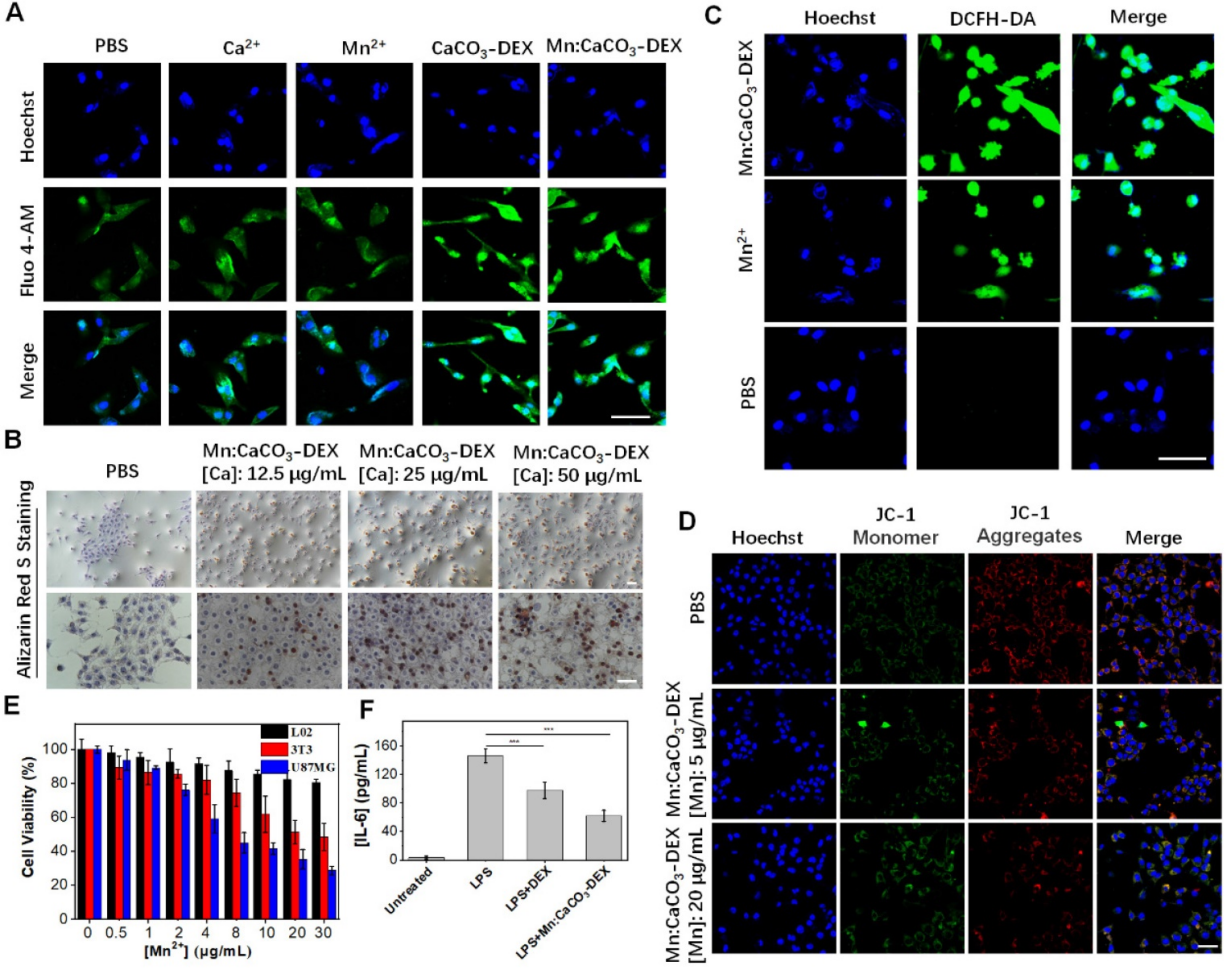
***In vitro* oxidative stress reaction and cell therapeutic effects. (A)** CLSM evaluation of cell uptake on U87MG cells incubated with PBS, Ca^2+^, Mn^2+^, CaCO_3_-DEX and Mn:CaCO_3_-DEX. The blue and green fluorescence indicate cell nucleus and an intracellular Ca^2+^ indicator, Fluo 4-AM, respectively (scale bar, 30 µm). **(B)** Identification of the formation of calcified nodules in U87MG cells with Alizarin Red S staining which show the calcified areas in red (scale bar, 100 µm). **(C)** Intracellular ·OH generation incubation with PBS, Mn^2+^ and Mn:CaCO_3_-DEX detected by DCFH-DA probe. The blue and green fluorescence indicate cell nucleus and DCFH-DA, respectively (scale bar, 30 µm). **(D)** CLSM observation on the changes in the mitochondrial membrane potential of U87MG cells after incubation with different concentration of Mn:CaCO_3_-DEX. The blue, red, and green colors indicate cell nucleus, and JC-1 J-aggregates and monomer, respectively (scale bar, 50 µm). **(E)** Cell viabilities of U87MG cells, L02 cells and 3T3 cells incubated with different concentration of Mn:CaCO_3_-DEX. **(F)** Secretion levels of pro-inflammatory cytokines (IL-6) changes in lipopolysaccharide- (LPS-) stimulated Raw264.7 cells after different treatment (***p < 0.001).

The concentration of free Ca^2+^ in the cytosol is generally less than 0.002 mM; it is a thousand or more times lower than that in the blood 19. This stimulation released Ca^2+^, which instantly increased the local Ca^2+^ levels in some cellular compartments with acidic environments. The evaluation on U87MG cells, a human glioma cell line, showed that the cellular Ca^2+^ signals (indicated by Ca^2+^ fluorescence probe, Fluo-4 AM) remained relatively stable when the U87MG cells were incubated with PBS, Mn^2+^ (10 μg/mL), and Ca^2+^ (25 μg/mL) (Figure 2A). In contrast, the cellular Ca^2+^ signals increased sharply when the cells were incubated with nanoparticles (CaCO_3_-DEX and Mn:CaCO_3_-DEX) because the nanoparticles could be endocytosed and not limited to the calcium ion channels (Figure 2A). Then, we also studied the distribution performance of Mn:CaCO_3_-DEX after they entered the cells through lysosome colocalization (Figure S9), the accumulation in mitochondrial (Figure S10) and thin-section cell TEM images (Figure S11). The co-localization of Mn:CaCO_3_-DEX and lysosomes indicated that nanoparticles can be located in lysosomes and mitochondrial. The Pearson's correlation coefficient of Ca^2+^ and lysosomes could reach to 0.65 ± 0.015 via ImageJ analysis. To confirm the uptake and disassociation of Mn:CaCO_3_-DEX, bio-TEM imaging of cell slices were performed. After 24 h incubation, Mn:CaCO_3_-DEX was observed in the lysosome (yellow arrow) and mitochondria (red arrow), and apparent degradations were observed (Figure S11). The abnormal boost growth of intracellular Ca^2+^ may lead to calcium overload and deactivate the calcium pump (Ca^2+^-ATP) (Figure S12), which may be accompanied by formation of calcification. Damage of the calcium outflow pathway could causes persistent calcium overload (Figure S12). The calcification of the cells was induced rapidly with vast accumulation of localized calcium ions. This was detected by Alizarin Red S staining (Figure 2B) 63. The formation of calcification nodules was observed significantly in treatment groups and the calcified area increased at higher concentrations of Mn:CaCO_3_-DEX. The obvious positive staining results proved that the released Ca^2+^ induced calcification profile.

The Trojan-horse strategy delivered ions into cells and disrupted the ion homeostasis 64-66. It affects cell functions, such as oxidative stress as well as mitochondrial dysfunction 67-69. To evaluate the oxidative stress response in cells, 2',7'-dichlorofluorescein diacetate (DCFH-DA; ROS fluorescence probe), was used to investigate the ·OH generation. Incubation of U87MG cells with Mn:CaCO_3_-DEX or Mn^2+^ free both induced significant fluorescent signal enhancement (Figures 2C, S13), proving the generation of intracellular ROS. Also, the fluorescence in U87MG cells incubated with Mn:CaCO_3_-DEX was much higher than that of the cells incubated with Mn^2+^ free at the same Mn concentration ([Mn]: 20 μg/mL). The rapid and abundant production of ROS could disrupt the original homeostasis state, which gave Mn:CaCO_3_-DEX the ability to oxidize biological molecules, leading to irreversible cell necrosis and apoptosis 45. The peroxidation of lipids (refers to the oxidative degradation of lipids) was elucidated via BODIPY-C11 (Figure S14A). Higher concentrations of Mn:CaCO_3_-DEX induced stronger fluorescence signals, and the average signal intensities increased by 7.4% (26.7 ± 0.4 of PBS VS 34.2 ± 5.5 of 5 μg/mL Mn) and 15.7% (42.5 ± 7.3 of 20 μg/mL Mn), respectively (Figure S14B). With the increasing of concentrations, the oxidized ratio could reach 98.5% (Figure S15).

The mitochondrial is participated in the apoptosis and death process of cells. This is also one of the major storage sites of Ca^2+^ in cells 70. The prolonged and high intracellular external ions levels would lead to mitochondrial dysfunction and then cell death. The mitochondrial membrane potential (MMP) plays a key role in the mitochondrial homeostasis, and a drop of MMP is a significant indicator of cell apoptosis, cell death and other pathologies. The 5,5',6,6'-tetrachloro-1,1',3,3'-tetraethylbenzimidazolyl-carbocyanine iodide (JC-1) assay kit was selected as a ratiometric probe to evaluate the MMP (ΔΨm) change, of which the red fluorescence shifted to green fluorescence after cell apoptosis. Compared with untreated cells, the red fluorescence decreased, but the green fluorescence increased after the cells were incubated with Mn:CaCO_3_-DEX (Figure 2D). ImageJ analysis showed much lower red-to-green ratios, indicating that the disturbed mitochondrial membrane potential could lead to cell damage (Figure S16). To verify the reason of mitochondrial damage, we also obtained the changes of JC-1 fluorescence signal after incubation with CaCO_3_-DEX. The decrease of red fluorescence indicated that the calcium overload could also induced mitochondrial damage (Figure S17).

Chronic inflammation plays an important role in the promotion of tumor proliferation 3. Macrophages could promote tumor growth and inflammation 24. DEX is a clinical anti-inflammatory agent and was evaluated for its anti-inflammatory effect via incubation of Raw264.7 with lipopolysaccharide (LPS) (100 ng/mL) to induce the pro-inflammatory cytokines 71. Significantly, Mn:CaCO_3_-DEX incubation induced a 2.4-fold decrease in inflammatory cytokine (IL-6) levels, which is higher than that of DEX treatment (1.5-fold) (Figure 2F).

After proving that the components are released and are destroyed, the cell therapeutic effects of Mn:CaCO_3_-DEX were studied by the standard methyl thiazolyl tetrazolium (MTT) cell viability assay. Satisfactorily, the Mn:CaCO_3_-DEX killed human tumor cells and showed negligible cytotoxicity on normal cells ( human L02 hepatocytes and mouse embryonic 3T3 fibroblasts) (Figure 2E). The half maximal inhibitory concentration (IC50) value was estimated as 3.4 μg Mn/mL, which was far lower than 3T3 cells (20.4 μg/mL) and L02 cells (no IC50 value in the concentration ranges). To level the combination, a combination index (CI) was calculated to assess the synergistic effects (CI < 1: synergism, CI > 1: antagonism, CI = 1: additive effect). In our study, the CI value was calculated to be 0.8, which indicated good combination of CDT and calcification effects. The results were further proved via live/dead (calcein-AM/propidium iodide (PI)) co-staining assays (Figure S18). The obvious red fluorescence signal indicated the Mn:CaCO_3_-DEX significantly inhibited cell proliferation. Lactate dehydrogenase (LDH) is an enzyme contained in the cytoplasm of living cells. It cannot penetrate the cell membrane under normal circumstances. However, after membrane permeability changes, LDH can be released into the medium, when target cells are attacked and damaged. Thus, the cytotoxicity was also estimated by comparing with positive controls. Significant LDH release was achieved, indicating that the function and structure of the cell membrane was destroyed (Figure S19). All of these results demonstrated that Mn:CaCO_3_-DEX could be a potential tumor-targeting chemical drug for cancer management, via HCO_3_^-^-indispensable Mn-mediated CDT, calcification and anti-inflammatory actions modulate the tumor environment and induce cell death.

To further explore the important role of pH-responsive release, we validated cell viability and the generation of ·OH after incubated with different buffer solutions. While incubation with free Ca^2+^ alone or with 1 mM H_2_O_2_, there was no significant cytotoxicity (Figure S20A). However, Mn^2+^ remarkably increased the cytotoxicity to tumor cells with the addition of H_2_O_2_ (1 mM) (Figure S20B). The cell survival rates reduced with stronger acidic environments (Figure S21A). We predicted that the stronger acidity of the incubation solution increased the released ions, resulting in more cell death. To prove this, we incubated cells with different concentrations of Mn^2+^, Ca^2+^, DEX and HCO_3_^-^ (according to the maximum release of Mn:CaCO_3_-DEX in different acidic environments) to verify the generation of ·OH and induced cytotoxicity Similar cell survivals (Figure S21B) and significantly increased fluorescence (Figure S22) proved that acidic-responsive drugs and ions release induced the cell death.

The 3D multicellular tumor spheroids (MTSs) were prepared to evaluated the penetration, distribution and therapeutic effect, which have major roles in tumor therapy research by virtue of their unique advantage of simulating the environment in tumor region. The penetration depth and fluorescence intensity increased obviously after incubating under acidic environment (pH 6.5) compared with the neutral environment (pH 7.4) (Figure S23), indicating that the acid decomposition capacity may facilitate their intercellular transportation. Furthermore, after 3 days of treatment, the growth of 3D MTSs were effectively inhibited (Figure S24). The 3D MTSs experiments provided a reference for the better prediction of their permeability and therapeutic effect in a solid tumor environment.

**Figure 3:**
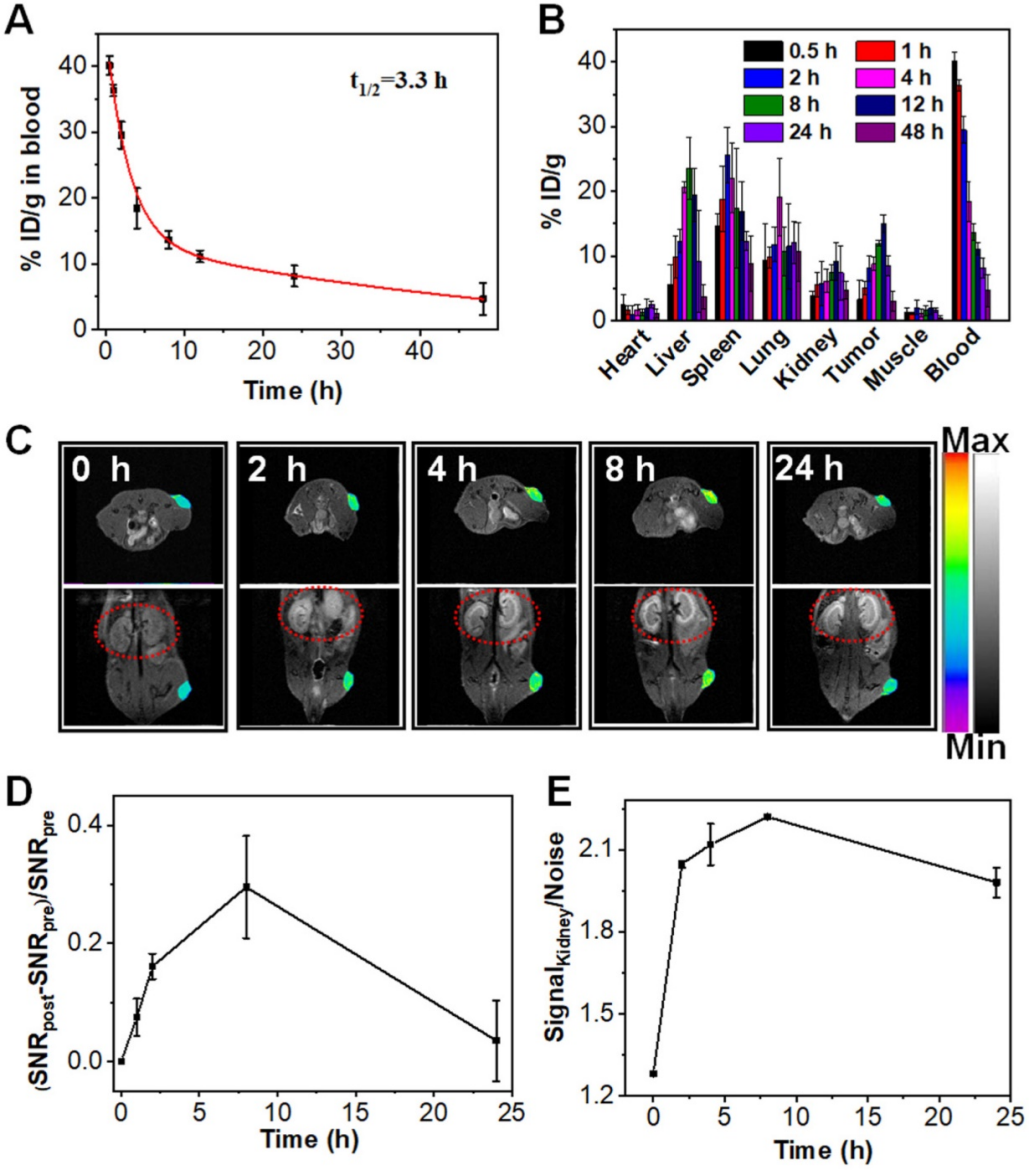
***In vivo* the pharmacokinetic and biodistribution evaluation of Mn:CaCO_3_-DEX. (A)** Blood circulation of Mn:CaCO_3_-DEX after intravenous injection. **(B)** Biodistribution of Mn (% injected dose (ID) Mn per gram of tissues) in main tissues and tumors after intravenous administration of Mn:CaCO_3_-DEX. **(C)** The T_1_-weighted MR images taken at different time points. **(D)** Quantification of the MR signals change in tumors within 24 h based on region of interest (ROI) analysis on images from panel (C). **(E)** Quantification of the MR signals change in kidney within 24 h based on ROI (red circles) analysis on images from panel (C).

The evaluation of the pharmacokinetics of Mn:CaCO_3_-DEX showed that the plasma terminal half-life of Mn:CaCO_3_-DEX was estimated to be 3.3 h (Figure 3A) The biodistribution of Mn:CaCO_3_-DEX in major organs and tumors was evaluated using U87MG tumor-bearing mice by measuring the content of Mn^2+^ using ICP-MS (Figure 3B). High accumulations of Mn^2+^ (15.0 ± 1.4 % ID/g) in tumors was achieved at 12 h post-injection; this showed excellent passive tumor targeting ability (Figure 3B). In the simulated acidic environment (pH 6.5, 1 mM H_2_O_2_), Mn:CaCO_3_-DEX degraded and released Mn^2+^ and exhibited excellent MR imaging ability (r_1_ = 5.8 mM^-1^ s^-1^) (Figure S25A). Positive contrast-enhancement were clearly observed around the injection sites in tumors, when Mn:CaCO_3_-DEX was injected directly into tumors of U87MG tumor-bearing mice (Figure S25B, C). Real-time monitoring of the distributions through T_1_-weighted MR imaging was important for *in vivo* therapy (Figure 3C). After intravenous injection with Mn:CaCO_3_-DEX, T_1_-weighted MR imaging of the tumors revealed clear positive-enhancement compared with the pre-injection (Figure 3C). The quantification of the strong positive MR signals in tumors (Figure 3D) proved that the Mn:CaCO_3_-DEX could be efficiently accumulated in tumors and gradually reduced to Mn^2+^, which was finally extracted by renal clearance (red circles in Figure 3C,E). Consistent with the MR imaging results, Mn:CaCO_3_-DEX could be obviously observed in the tumor region by Bio-TEM images of tumor tissues (Figure S26).

To detect the generation of ·OH in the tumor through the treatment of Mn:CaCO_3_-DEX, the tumor-bearing mice were intravenously or intratumoral injected with Mn:CaCO_3_-DEX ([Mn]: 2 mg/kg, [DEX]: 4 mg/kg, [Ca]: 5 mg/kg) for fluorescence imaging. The results indicated that Mn:CaCO_3_-DEX could produce a large number of ·OH, inducing cellular oxidative stress (Figure S27).

**Figure 4:**
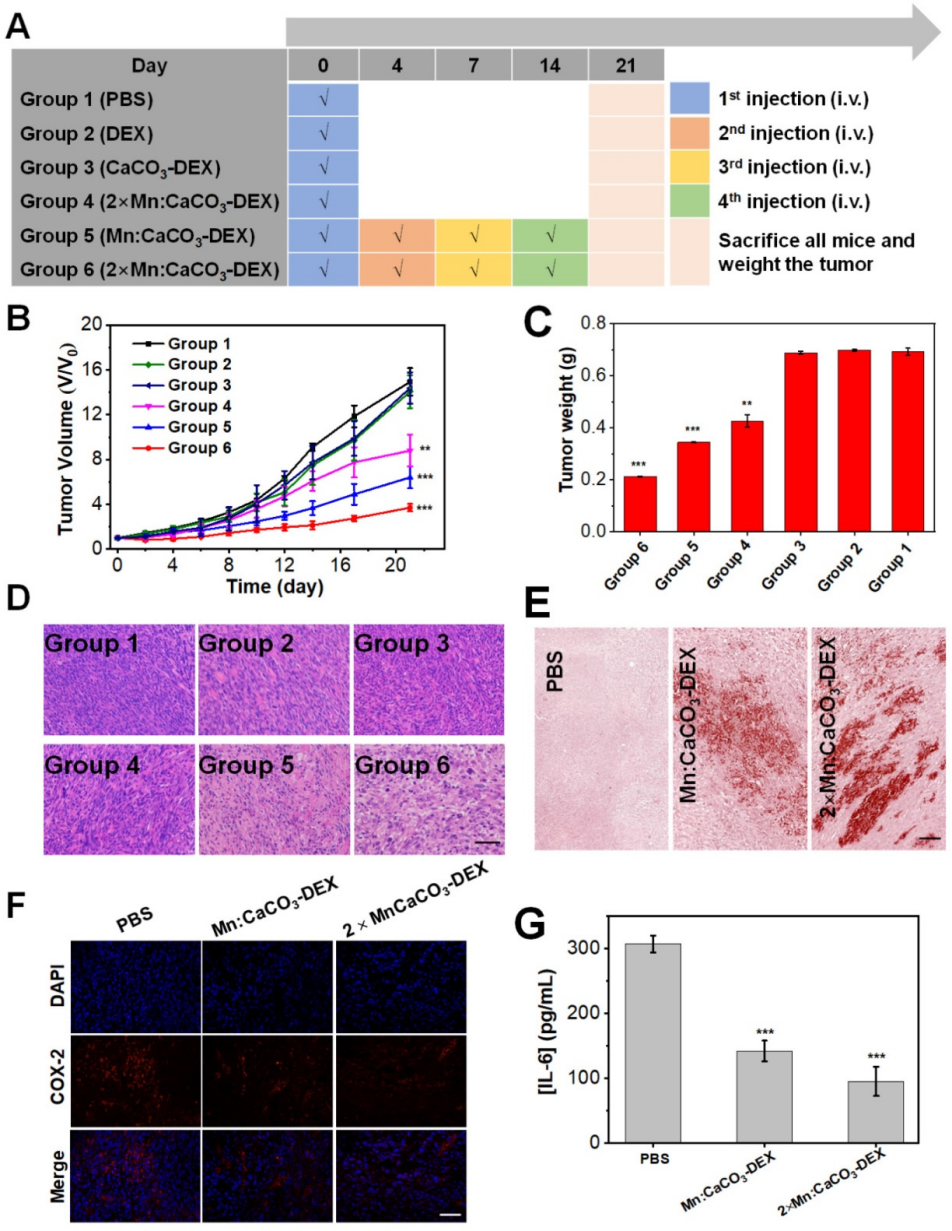
***In vivo* antitumor efficacy for subcutaneous tumor. (A)** Schematic illustration of the administration design. Groups 1, 2, 3, 4, 5 and 6 were used to represent PBS; DEX (4 mg/kg, one dose); CaCO_3_-DEX ([DEX]: 4 mg/kg, [Ca]: 5 mg/kg, one dose); 2×Mn:CaCO_3_-DEX ([Mn]: 4 mg/kg, [DEX]: 8 mg/kg, [Ca]: 10 mg/kg, one dose); Mn:CaCO_3_-DEX ([Mn]: 2 mg/kg, [DEX]: 4 mg/kg, [Ca]: 5 mg/kg, four doses); 2×Mn:CaCO_3_-DEX ([Mn]: 4 mg/kg, [DEX]: 8 mg/kg, [Ca]: 10 mg/kg, four doses). **(B)** Tumor growth curves of U87MG tumor-bearing mice exposed to different formulations after the 21-day treatment period (**p < 0.01; ***p < 0.001). **(C)** Final tumor weights of U87MG tumor-bearing mice exposed to different formulations after the 21-day treatment period. **(D)** Hematoxylin & eosin (H&E)-stained tumor sections from U87MG tumor-bearing mice after the 21-day different treatment period (scale bar: 100 µm). **(E)** Alizarin Red S staining of tumor tissue sections from U87MG tumor-bearing mice after the 21-day different treatment period. PBS; Mn:CaCO_3_-DEX ([Ca]: 5 mg/kg, four doses); 2×Mn:CaCO_3_-DEX ([Ca]: 10 mg/kg, four doses) (scale bar: 100 µm). **(F)** COX-2 immunofluorescence staining of U87MG tumor tissues after the 21-day different treatment period. PBS; Mn:CaCO_3_-DEX ([DEX]: 4 mg/kg, four doses); 2×Mn:CaCO_3_-DEX ([DEX]: 8 mg/kg, four doses), The blue and red fluorescence indicate cell nucleus and COX-2, respectively (scale bar:100 µm). **(G)** Secretion levels of pro-inflammatory cytokines (IL-6) in tumors after different treatment. PBS; Mn:CaCO_3_-DEX ([DEX]: 4 mg/kg, four doses); 2×Mn:CaCO_3_-DEX ([DEX]: 8 mg/kg, four doses). (***p < 0.001).

After confirming the in-situ generation of ions bombs (Ca^2+^, Mn^2+^, and HCO_3_^-^) and release of drug (DEX), The *in vivo* therapeutic effects (enhanced CDT, Ca^2+^ overloading) and anti-inflammatory were evaluated in both subcutaneous and orthotopic tumor models. When the tumor volume reached 60 mm^3^, the U87MG subcutaneous tumor-bearing mice were randomly divided into 6 groups (n=5) (Figure 4A): (Group 1) PBS; (Group 2) DEX (4 mg/kg, one dose); (Group 3) CaCO_3_-DEX ([DEX]: 4 mg/kg, [Ca]: 5 mg/kg, one dose); (Group 4) 2×Mn:CaCO_3_-DEX ([Mn]: 4 mg/kg, [DEX]: 8 mg/kg, [Ca]: 10 mg/kg, one dose); (Group 5) Mn:CaCO_3_-DEX ([Mn]: 2 mg/kg, [DEX]: 4 mg/kg, [Ca]: 5 mg/kg, four doses); and (Group 6) 2×Mn:CaCO_3_-DEX ([Mn]: 4 mg/kg, [DEX]: 8 mg/kg, [Ca]: 10 mg/kg, four doses). The curves in 21-day treatments showed Groups 4-6 had obviously inhibited tumor growth. They had lower tumor weights and smaller tumor sizes compared to Groups 1-3 (Figure 4B, C). We also obtained photographs of mice in different groups during the treatment to visually assess the effect of treatment (Figure S28). To quantify the treatment effect, the tumor growth inhibition indices (TGIs) were calculated in Equation 1:


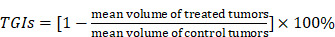

(1)

The TGI values of Group 6 (75.2%) was significantly higher than the low concentration treatment group (Group 5: 57.0%) and low dose treatment group (Group 4: 41.1%). These results proved that there was a close relation between the therapeutic effect and the administration dosage and interval.

To highlight the necessity of nanomaterials in synergistic oncotherapy, the group MnCl_2_+ CaCO_3_ +DEX ([Mn]: 4 mg/kg, [DEX]: 8 mg/kg, [Ca]: 10 mg/kg, four doses) for tumor therapy was also validated. As shown in Figure S29, the therapeutic effect was not significant.

The hematoxylin & eosin (H&E) staining results of the tumors (Figure 4D) showed significant damage in Group 6 and proved tumor apoptosis and necrosis. The tumor sections were also stained with Alizarin Red S staining, indicating the generation of calcification (Figure 4E). COX-2 expression and the inflammatory cytokine (IL-6) levels in tumors decreased sharply in the successful treatment groups (Figure 4F, G) 72-73. These results suggest that the superior therapeutic effects in Group 6 were attributed to the combination of anti-inflammatory, cell calcification and HCO_3_^-^-enhanced Mn-mediated CDT. Most importantly, during the 21-day therapeutic period, the mice body weight (Figure S30) did not decrease. The H&E staining images of the major organs (Figure S31) after treatments caused no apparent detrimental effects on systemic health, suggesting minimal toxicity to normal cells. Complete serum chemical analysis showed that there were no significant changes after treatment (Figure S32), indicating liver and kidney function of the mice remained normal. After entry into a body, nanoparticles tend to accumulate in liver and lead to hepatic damage, which can be reduced greatly by enhancing Kupffer cell survival. Cytotoxicity *in vitro* proved that Mn:CaCO_3_-DEX was nontoxic to Kupffer cell line (Figure S33A). Immunostaining for liver tissues after Mn:CaCO_3_-DEX treatment showed Mn:CaCO_3_-DEX could not decrease Kupffer cell numbers (Figure S33B-D).These results demonstrated that Mn:CaCO_3_-DEX had good biocompatibility, low systemic toxicity and satisfactory tumor suppressive effects.

**Figure 5:**
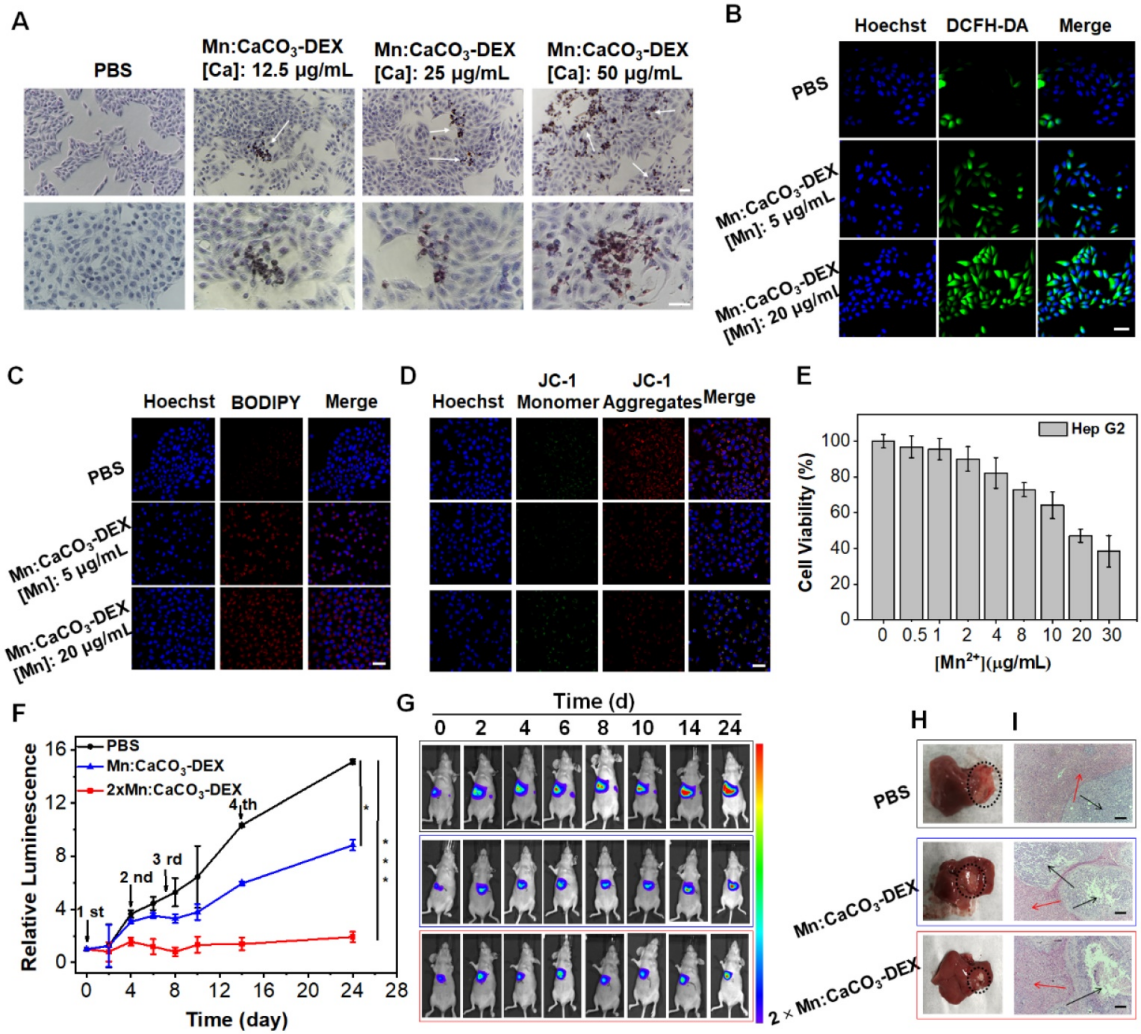
** The antitumor efficiency for human hepatocellular carcinoma (HepG2) cells and Orthotopic Hepatic Tumors**. **(A)** Identification of the formation of calcified nodules in HepG2 cells with Alizarin Red S staining, which show the calcified areas in red, respectively (scale bar, 100 µm). **(B)** Intracellular ·OH generation detected by DCFH-DA probe. The blue and green fluorescence indicate cell nucleus and DCFH-DA, respectively (scale bar, 50 µm). **(C)** CLSM observation on the intracellular distribution of lipoperoxides in HepG2 cells after incubation with PBS and different concentration of Mn:CaCO_3_-DEX for 24 hours. The red fluorescence is the lipid ROS in cells and membranes after the staining with BODIPY-C11, respectively (scale bar, 50 µm). **(D)** CLSM observation on the changes in the mitochondrial membrane potential of HepG2 cells after incubation with different concentration of Mn:CaCO_3_-DEX. The blue, red, and green colors indicate cell nucleus, and JC-1 J-aggregates and monomer, respectively (scale bar, 50 µm). **(E)** Cell viabilities of HepG2 cells incubated with different concentration of Mn:CaCO_3_-DEX. **(F)** Relative luminescence intensity changes based on the Bioluminescence images (BLI) (*p < 0.05; ***p < 0.001). **(G)** BLI changes of different treatments on days 0-24. **(H)** Photographs of representative tumors taken from orthotopic hepatic tumor-bearing mice after various treatments. **(I)** H&E-stained tumor sections from tumor-bearing mice after various treatments (scale bar: 100 µm).

We further evaluated the anticancer activity of Mn:CaCO_3_-DEX on an orthotopic hepatocellular carcinoma (HepG2). The Alizarin Red S staining results demonstrated the formation of calcification accompanied by the uptake of Mn:CaCO_3_-DEX (Figure 5A). DCFH-DA staining and BODIPY-C11 staining showed significant enhancement of the fluorescence (Figure 5B, C), indicating the efficient generation of ROS and oxidation of lipids. The JC-1 staining results and the release of LDH proved the dysfunction of mitochondria and breakage of cell membranes (Figures 5D, S34). These dysfunctions resulted in low cell viability after treatment of Mn:CaCO_3_-DEX (Figure 5E).

The therapeutic efficiency of Mn:CaCO_3_-DEX was further studied on orthotopic liver tumor models. After confirming the establishment of tumors by bioluminescence imaging (BLI), the tumor-bearing mice were divided randomly into 3 groups (n = 5/group): (1) PBS; (2) Mn:CaCO_3_-DEX ([Mn]: 2 mg/kg, [DEX]: 4 mg/kg, [Ca]: 5 mg/kg); and (3) 2×Mn:CaCO_3_-DEX ([Mn]: 4 mg/kg, [DEX]: 8 mg/kg, [Ca]: 10 mg/kg). Four injections were administrated intravenously on 0, 2, 7 and 14 days. During the treatment, the orthotopic hepatic tumor growth was monitored by BLI every 2 days. The BLI of the injection of Mn:CaCO_3_-DEX increased slowly, and the results indicated significant inhibition effects after 24 day of treatment. Versus baseline, a 15.1-fold luminescence enhancement was achieved in PBS group. In contrast, a 8.8-fold enhancement was achieved in the Mn:CaCO_3_-DEX group. Significantly, only a 1.9-fold signal enhancement was seen in the 2×Mn:CaCO_3_-DEX group (Figure 5F, G). The photographs of the tumors in excised livers on day 24 clearly showed the size and position of the orthotopic hepatic tumors (Figure 5H, blank circles), confirming the efficient inhibition. More importantly, H&E staining of orthotopic hepatic tumors (Figure 5I, black arrows) showed severely damaged structural disruptions and pathological changes, indicating that high dose of Mn:CaCO_3_-DEX caused cancer cell apoptosis. The staining of Alizarin Red S and COX-2 immunohistochemistry again proved the generation of calcification and suppression of inflammation (Figure S35). Furthermore, there was no change in normal liver tissues (Figure 5i, red arrows). The fluctuation of body weight was negligible in all mice (Figure S36). Overall, these results demonstrated that Mn:CaCO_3_-DEX had good biocompatibility, and could be an excellent ion drug for precise orthotopic liver cancer therapy.

## Conclusion

In summary, a responsive and biodegradable biomineral—Mn-doped calcium carbonate-dexamethasone phosphate (DEX) nanoparticles (Mn:CaCO_3_-DEX)—was reported. Under the acidic environment, Mn:CaCO_3_-DEX decomposed to form ions (Mn^2+^, Ca^2+^, CO_3_^2-^) and release anti-inflammatory and antitumor drug (DEX). The released ions amplified the oxidative stresses via the *in situ* self-supplying HCO_3_^-^-enhanced Mn-mediated CDT. This modulated the tumor environment to inhibit tumor growth. At the same time, the released Ca^2+^ destroyed the intracellular and extracellular ionic gradients to amplify tumor oxidative stress and Ca-overload cell apoptosis and calcification. Furthermore, the released anti-inflammatory drug, DEX, can alleviate the inflammatory environment. The investigations *in vitro* and *in vivo* demonstrated that the synergistic oncotherapy enables synergistic amplification of tumor oxidative stress, reduces inflammation, and induces Ca-overload cell apoptosis to effectively inhibit the growth of subcutaneous tumors. There is almost no influence on normal cells. Overall, Mn:CaCO_3_-DEX is an excellent potential agent for precise orthotopic tumor management by simple selecting the coordination pairs between functional organic molecules and metal ions to fit alternate imaging and therapeutic roles.

## Supplementary Material

Supplementary methods and figures.
