# Supplementary material for: Ion drugs for precise orthotopic tumor management by in situ the generation of toxic ion and drug pools
Source: Theranostics. 2022 Jan 1;12(2):734–46. doi: 10.7150/thno.66468 (PMC8692900; doi:10.7150/thno.66468)
Supplement: Supplementary file 1 — Supplementary methods and figures. [file thnov12p0734s1.pdf]

Supporting Information for

Ion drugs for precise orthotopic tumor management by in situ the generation of toxic ion and drug pools

Yushuo Feng, Ruixue Qin, Lihua Xu, Xiaoqian Ma, Dandan Ding, Shi Li, Lei Chen, Yaqing Liu, Wenjing Sun, Hongmin Chen*

Experimental Procedures

Materials and Characterization.

Poly (allylamine hydrochloride) (PAH), hydrogen peroxide (H_2O_2), methylene blue (MB), 4-nitrophenyl chloroformate 3-(4,5-dimethylthiazol-2-yl)-2,5diphenyltetrazolium bromide (MTT) and AM/PI dual-staining kit were purchased from Sigma-Aldrich. dexamethasone sodium phosphate purchased from Energy Chemical. Calcium chloride tetrahydrate ($CaCl_2 \cdot 2H_2O$) and ammonia bicarbonate (NH_4HCO_3) were purchased from Sinopharm Chemical Reagent CO, Ltd, China. Fluo-4 AM, 2',7'-Dichlorodihydrofluorescein diacetate (DCFH-DA), lactate dehydrogenase (LDH) Assay Kit and Lyso-Tracker Red were purchased from Shanghai Beyotime Biotechnology Co., Ltd. JC-1 were purchased from MedChemExpress. BODIPY™ 581/591 C11 (Lipid Peroxidation Sensor) were purchased from thermo fisher scientific.

Transmission electron microscopy (TEM) was carried out on a JEOL 1200EX transmission electron microscope. Absorption spectra were recorded using a UV–vis spectrometer (Agilent Cary60, USA). The crystal structure was analyzed by a PANalytical X’Pert PRO powder X-ray diffractometer (XRD) with Cu Kα1 radiation. The fluorescence images of cells were taken on a laser scanning confocal microscopy (Olympus FV1200, Japan). MR imaging was conducted on a 9.4 T small animal MRI system (Bruker, Germany).

Synthesis of Mn-doped $CaCO_3$-Dexamethasone (Mn:$CaCO_3$-DEX) Nanoplatform.

Calcium carbonate-dexamethasone ($CaCO_3$-DEX) nanoparticles were synthesized by adopting the onepot gas diffusion process with modifications.

Briefly, 50 mg of $CaCl_2 \cdot 2H_2O$ was mixed with 2 mg, 4 mg or 6 mg of dexamethasone sodium phosphate (DEX), and then dissolved in a beaker containing 50 mL of ethanol. At room temperature, the mixture was stirred 1 h and placed in a sealed container with NH_4HCO_3. After 2, 6 or 8 h, the white calcium carbonate-dexamethasone nanoparticles were collected and purified with ethanol by repeated centrifugation at 5000 rpm. To prepare Manganese-doped $CaCO_3$-DEX (Mn:$CaCO_3$-DEX) nanoparticles, the $CaCO_3$-DEX solution was mixed with ethanol solution containing $MnCl_2 \cdot 2H_2O$ (0.1 mg/mL). After stirred 3 h, the Mn:$CaCO_3$-DEX were centrifuged and washed three times with ethanol to remove the unbound manganese ions. To improve water solubility, the Mn:$CaCO_3$-DEX nanoparticles were modified with PAH (5 mg/mL). Finally, the obtained nanoparticles were dried at 60 ^oC.

The loading efficiency was evaluated by the Equations.

The absorbance of DEX was measured using a spectrometer in different concentration. And the drug encapsulation efficiency was obtained by quantifying the absorption of $CaCO_3$-DEX (241 nm). Loading capacity (LC) was calculated using eq 1:

$$LC\% = \frac{weight\ of\ loading\ DEX}{weight\ of\ CaCO_3 - DEX} \times 100$$

Entrapment efficiency (EE) was calculated using eq 2:

$$EE\% = \frac{weight\ of\ loading\ DEX}{weight\ of\ DEX_{added}} \times 100$$

Degradation and drug release studies.

Mn:$CaCO_3$-DEX was incubated with PBS solution for different durations: (a) pH 7.4, (b) pH 6.5, (c) pH 6.5 with 1 mM H_2O_2 and (d) pH 4.0. At the given time points, the solutions were centrifuged to collect the precipitation and supernatant. The release of Mn^{2+}, Ca^{2+} and DEX were respectively measured by T_1-MRI (1.5 T), ICP-OES and the absorbance at 241 nm in the collected

supernatant. The precipitation was also measured by the TEM to prove the change of nanoparticles morphology. The generation of CO_2 bubbles with different concentration of Mn:$CaCO_3$-DEX were detected by an ultrasound imaging system with the acidic environment (pH 6.5 + H_2O_2).

Detected the generation of ·OH in solution.

Mn:$CaCO_3$-DEX ([Mn]: 50 μg/mL) was incubated with or without containing $NaHCO_3$ (1 mM, the highest possible concentration that released from Mn:$CaCO_3$-DEX matrix) for 1 h in acidic environment (pH 6.5). After centrifugation, 10 μg/mL MB and 5 mM H_2O_2 were added into the supernatant. After 30 min, the absorbance of MB was measured.

Cell culture.

Human glioblastoma (U87MG) cells, human hepatocellular carcinoma (Hep G2) cells, the normal human hepatocytes (L02), mouse embryo fibroblasts (3T3), mouse mononuclear macrophages (Raw264.7) and Kupffer cells were cultured at 37 °C within 5% CO_2.

Cell uptake.

An observation dish was selected to obtain confocal images. U87MG cells were seeded in observation dish at a density 10^5 cells for 24 h. Then the cells were incubated with PBS, Mn^{2+} (10 μg/mL), Ca^{2+} (25 μg/mL), $CaCO_3$-DEX ([Ca]: 25 μg/mL) and Mn:$CaCO_3$-DEX ([Ca]: 25 μg/mL) for 24 h. After washing with PBS for three times, the cells were successively incubated with Fluo4-AM (2 μM) and hoechst33342 (5 μg/mL). The cells were imaged by a laser scanning confocal fluorescence microscope.

Co-localization of Mn:$CaCO_3$-DEX with lysosome.

To prove the intracellular distribution, Hep G2 cells were also seeded in observation dish at a density 10^5 cells for 24 h, and incubated with Mn:$CaCO_3$-DEX ([Ca]: 25 μg/mL) in the dark for 24 h. After washing with PBS for three times, the cells were stained with hoechst33342 (5 μg/mL) and lyso-tracker (2 μM). The stained cells were imaged by a laser scanning confocal fluorescence microscope.

Identification of the Ca^{2+} accumulate in mitochondria

To prove the Ca^{2+} accumulate in mitochondria, U87 cells were seed in observation dish at a density 10^5 cells for 24 h, and incubated with $CaCO_3$-DEX ([Ca]: 25 μg/mL) for 24 h. After washing with PBS for three times, the cells were stained with hoechst33342 (5 μg/mL) and Rhod-2-AM (2 μM). The stained cells were imaged by a laser scanning confocal fluorescence microscope.

Identification of subcellular organelle morphology and the material distribution by bio-TEM.

Hep G2 cells were cultivated with Mn:$CaCO_3$-DEX ([Ca]: 25 μg/mL) at 37°C for 24 h and then fixed by glutaraldehyde fixing solution (2.5%) at 4°C. The cells were then dehydrated, embedded and sliced and imaged by a bio-TEM.The uptake of Mn:$CaCO_3$-DEX under 3D multicellular tumor spheroids (MTSs).

Hep G2 cells (1000 cells per well) were seeded into 96-wells plate to form MTSs. In this experiment, the Mn:$CaCO_3$-DEX-Cy5.5 ([Ca]: 25 μg/mL) were added to the dishes of the MTSs for 24 h. The uptake and size distribution after co-incubating with nanoparticles were captured by a laser scanning confocal fluorescence microscope.

Oxidative stress assessment.

For reactive oxygen observation, U87MG cells and Hep G2 cells were respectively seeded in observation dish at a density 10^5 cells, and incubation PBS, Mn^{2+} (20 μg/mL) and Mn:$CaCO_3$-DEX ([Mn]: 20 μg/mL) for 24 h. After being washed with PBS, the cells were incubated with hoechst33342 (5 μg/mL) and DCFH-DA (5 μM) for 30 min. Subsequently, the cells were washed with PBS for three times and the fluorescence images were acquired by a laser scanning confocal fluorescence microscope.

To prove acid-activatable oxidative stress-inducing anticancer therapy, Hep G2 cells were also seeded in observation dish at a density 10^5 cells for 24 h, and incubated with Mn:$CaCO_3$-DEX ([Mn]: 20 μg/mL) with different buffer solutions (pH 4.0, 6.5 and 7.4) in the dark for 24 h. After being washed with PBS, the cells were incubated with hoechst33342 (5 μg/mL) and DCFH-DA (5 μM) for 30 min. Subsequently, the cells were washed with PBS for three times and the fluorescence images were acquired by a laser scanning confocal fluorescence microscope.

To evaluate the intracellular lipid peroxidation levels, BODIPY C11 was selected as the testing kits. U87MG cells and Hep G2 cells were respectively seeded observation dish at a density 10^5 cells for 24 h. Then, the cells were treated with Mn:$CaCO_3$-DEX ([Mn]: 5 and 20 μg/mL) for 24 h. After the incubation, the cells in observation dish were washed with PBS for three times and stained with hoechst33342 (5 μg/mL) and BODIPY C11 (5 μM), and then obtained the fluorescence images.

For lactate dehydrogenase (LDH) release assay, U87MG cells and Hep G2 cells were respectively seeded in 96-well plates (10^4 cells per well) and incubation for 24 h prior to the experiment. The cells were treated with Mn:$CaCO_3$-DEX ([Mn]: 5 and 20 μg/mL) for 24 h. The LDH release was tested according with the vendor's protocol. Briefly, the cell-only group were mixed

with LDH releasing agent to acquire the maximum LDH release. Then, the supernatant (120 μL) in DMEM-only group, cell-only group, the maximum LDH release group and other experiment group was collected and tested.

To monitor the changes of mitochondrial membrane potential, JC-1 (a fluorescent lipophilic carbocyanine dye) were selected as detection probe. U87MG cells and Hep G2 cells were respectively seeded in observation dish at a density 10^5 cells for 24 h. The cells were treated with Mn:$CaCO_3$-DEX ([Mn]: 5 and 20 μg/mL). After incubation for 24 h, the cells were washed with PBS for three times and stained with hoechst33342 (5 μg/mL) and JC-1 (2.5 μM). After washing with PBS for three times, the fluorescence images were acquired on an Olympus FV1200 laser scanning confocal microscope.

The detection for the formation of calcified nodules.

For Alizarin Red S staining, U87MG cells and Hep G2 cells were respectively seeded in 12-well plates for 24 h. The cells were treated with Mn:$CaCO_3$-DEX ([Ca]: 12.5, 25 and 50 μg/mL) for another 24 h. After removal the DMEM, the cells were washed with PBS (without calcium salt) for three times and fixed with 4% paraformaldehyde. The staining for the formation of calcified nodules was tested according with the vendor’s protocol. Briefly, for Alizarin Red S staining, the cells were stained with Alizarin Red S staining solution for 10 min and washed with PBS (without calcium salt) for five times. Then, the cells were observed and imaged under white light by the inverted fluorescence microscope. Finally, the cells were observed and imaged under white light by the inverted fluorescence microscope.

The inhibition of inflammatory cytokines secreting.

For the inhibiting assay, Raw 264.7 cells were seeded in 96-well plates for 24 h. After being stimulated with 100 ng/mL lipopolysaccharides (LPS) for 24 h,

the cells were incubated with DEX (50 μg/mL) and Mn:$CaCO_3$-DEX ([DEX]: 50 μg/mL) for 24 h. The expression of IL-6 was quantified by the ELISA kit.

In vitro therapy effect.

To evaluate the in vitro therapy effect, U87MG cells, L02 and 3T3 were seeded in 96-well plates for 24 h. Then, the cells were incubated with different concentrations of Mn:$CaCO_3$-DEX for 24 h. The cell viability was measured by the MTT assay.

The cell death was also detected by the live/dead assay investigation. U87MG cells were seeded in 6-well plates at a density 10^5 cells for 24 h. Then the cells were incubated DEX ([DEX]: 50 μg/mL), $CaCO_3$-DEX ([Ca]: 62.5 μg/mL and [DEX]: 50 μg/mL), Mn:$CaCO_3$-DEX ([DEX]: 50 μg/mL, [Mn]: 25 μg/mL and [Ca]: 62.5 μg/mL) for 24 h. Calcein-AM (ex/em:490/515 nm) and PI (ex/em: 517/617 nm) were used to stain the live and dead cells. After being washed with PBS, the cells were digested and resuspended in the PBS solution. The images were obtained by the fluorescence microscope.

To further explore the role of pH-responsive drug release, we validated cell viability after incubated with Mn:CaCO3-DEX ([Mn]: 10 μg/mL; ([Ca]: 25 μg/mL; ([DEX]: 20 μg/mL) in different buffer solutions (pH 7.4, 6.5 and 4.0). The cell viability was measured by the MTT assay. Then, the cells were also incubated cell with different concentration of Mn^{2+}, Ca^{2+}, DEX and HCO_3^- (according to the maximum release of Mn:$CaCO_3$-DEX in different acidic environment) to determine its activity by MTT. ((Maximum release of Mn:$CaCO_3$-DEX ([Mn]: 10 μg/mL; [Ca]: 25 μg/mL; [DEX]: 20 μg/mL) in different buffer solutions). Group 1: the aximum release in pH 7.4 ([Mn]: 4.0 μg/mL; [Ca]: 9.0 μg/mL; [DEX]: 3.8 μg/mL; [HCO_3^-]: 13.1 μg/mL); Group 2: the aximum release in pH 6.5 ([Mn]: 6.7 μg/mL; [Ca]: 19.7 μg/mL; [DEX]: 11.3 μg/mL; [HCO_3^-]: 28.7 μg/mL); Group 3: the aximum release in pH 4.0; [Mn]: 9.2 μg/mL;

[Ca]: 23.0 µg/mL; [DEX]: 12.5 µg/mL; [HCO_3^-]: 33.58 µg/mL).

To evaluate the ability of the drug to fully inhibit the growth of the spheroid, a Mn concentration of 50 µg/mL (Mn:$CaCO_3$-DEX) was employed. The size was captured and recorded.

Biodistribution and blood circulation.

U87MG tumor-bearing mice were intravenously injected with Mn:$CaCO_3$-DEX ([Mn]: 2 mg/kg, [DEX]: 4 mg/kg, [Ca]: 5 mg/kg). The major tissue (heart, livers, spleen, lungs, kidneys, tumor, muscle) and blood samples (orbital venous plexus) were collected at different time points (0.5, 1, 2, 4, 8, 12, 24 and 48 h) and dissolved in HNO_3-H_2O_2 solutions. The Mn content were tested by inductively couple plasma mass spectrometry (ICP-MS)

Tumor environment triggered Mn:$CaCO_3$-DEX decomposition.

To evaluate the generation of Mn^{2+} and the contrast effect, Mn:$CaCO_3$-DEX ([Mn]: 50 µg/mL, 20 µL) was injected intratumorally. MR imaging were obtained on a 9.4 T MRI small animal MRI system (Bruker, Germany) at the different post-injection time points (10, 20, 30, 60, 120, 180 and 240 min). T_1-weighted MRI were measured using a spin echo multiple slice sequence, and the parameters were the following: T_1 imaging: TR =1000.0 ms; TE = 8.5 ms; matrix = 256 × 256.

In vivo imaging.

Tumor-bearing mice were intravenously injected with Mn:$CaCO_3$-DEX ([Mn]: 2 mg/kg, [DEX]: 4 mg/kg, [Ca]: 5 mg/kg) for in vivo imaging. MR imaging was conducted on a 9.4 T MRI small animal MRI system (Bruker, Germany) at the different post-injection time points. The signal changes of tumor were recorded. T1-weighted MRI were measured using a spin echo multiple slice

sequence, and the parameters were the following: T1 imaging: TR =1000.0 ms; TE = 8.5 ms; matrix = 256 × 256; multislice spin−echo sequence with a field of view of 40 × 40 mm; slice=15; slice thickness= 1mm × 1 mm.

The detection for the generation of ·OH in the tumor

When the tumor reached 60 mm^3 in average volume, the mice were randomly divided into 4 groups：Group1: control: without any treatment; Group2: PBS: DCFH-DA was injected into the tumor after PBS injection for 8 h. Group 3: DCFH-DA was injected into the tumor after Mn:$CaCO_3$-DEX (i.v.) injection for 8 h; Group 4: Mn:$CaCO_3$-DEX (i.v.) DCFH-DA was injected into the tumor after Mn:$CaCO_3$-DEX (i.t.) injection for 10 min. (Mn:$CaCO_3$-DEX ([Mn]: 2 mg/kg, [DEX]: 4 mg/kg, [Ca]: 5 mg/kg))

In vivo antitumor efficacy for subcutaneous tumors.

When the tumor reached 60 mm^3 in average volume, the mice were randomly divided into 6 groups: (Group 1) PBS; (Group 2) DEX (4 mg/kg, one dose); (Group 3) $CaCO_3$-DEX ([DEX]: 4 mg/kg, [Ca]: 5 mg/kg, one dose); (Group 4) 2×Mn:$CaCO_3$-DEX ([Mn]: 4 mg/kg, [DEX]: 8 mg/kg, [Ca]: 10 mg/kg, one dose); (Group 5) Mn:$CaCO_3$-DEX ([Mn]: 2 mg/kg, [DEX]: 4 mg/kg, [Ca]: 5 mg/kg, four doses); (Group 6) 2×Mn:$CaCO_3$-DEX ([Mn]: 4 mg/kg, [DEX]: 8 mg/kg, [Ca]: 10 mg/kg, four doses). The body weight and tumor size of the mice were recorded for the next 21 days, and the tumor volume was calculated according to the formula $V=L\times W^2/2$, where L and W denoted the maximum tumor length and width. The relative tumor volume of each mouse was acquired by dividing by the initial tumor volume Tumor volumes and body weight were also recorded. After 21 days, the mice were euthanized and the collected tumor and major organ were kept for H&E staining.

Biosafety analysis.

BALB/c nude mice were intravenously injected with Mn:$CaCO_3$-DEX ([Mn]: 4 mg/kg, [DEX]: 8 mg/kg, [Ca]: 10 mg/kg; four doses). Blood samples were collected on day 0, day 7, day 14 and day 21. AST, aspartate transaminase; ALT, alanine transaminase; ALP, A Lkaline Phosphatase and UREA, blood urea nitrogen. were measured using an Automatic Biochemistry Analyzer (Mindray, BS-220).

To evaluate the hepatotoxicity, Kupffer cells were seeded in 96-well plates for 24 h. Then, the cells were incubated with different concentrations of Mn:$CaCO_3$-DEX for 24 h. The cell viability was measured by the MTT assay. F4/80 was used as the marker of kupffer cells to perform Immunofluorescence staining for liver tissue.

Histopathologic evaluation.

After the sacrifice of the mice at day 21, the tumor of Gorup1, 5 and 6 were assessed global inflammation, through expression of inflammatory factors (IL-6) and the immunofluorescence stained with cyclooxygenase-2 (COX-2). To evaluate the formation of calcified nodules by Mn:$CaCO_3$-DEX, the tumor of Gorup1, 5 and 6 were also be stained by Alizarin Red S.

The antitumor efficiency for orthotopic hepatic tumors.

Female nude mice (BALB/C, 18−20 g) purchased from the Animal Care and Use Committee of Xiamen University, were used for assessing the treatment effect. Briefly, mice were anesthetized with 5% chloral hydrate, and then 20 μL Hep G2/Luc cells (5×10^6 per mouse) were injected into the right liver lobe by a laparotomy. About 7 days later, bioluminescence images performed with IVIS Lumina II after intraperitoneal injection of fluorescein substrate to screen out tumor-bearing mice for in vivo therapy. The tumor formation rate was 80%.

After confirming tumor establishment, the tumor-bearing mice were divided randomly into 3 groups (n = 5/group): (Group 1) PBS; (Group 2) Mn:$CaCO_3$-DEX ([Mn]: 2 mg/kg, [DEX]: 4 mg/kg, [Ca]: 5 mg/kg); (Group 3) 2×Mn:$CaCO_3$-DEX ([Mn]: 4 mg/kg, [DEX]: 8 mg/kg, [Ca]: 10 mg/kg). During the entire treatment period, body weights and in vivo bioluminescence images were recorded every 2 days after the different treatments. All mice were euthanized on day 24 after tumor therapy, then the tumors were taken out and photographed. The excised tumors were collected for standard H&E staining, Alizarin Red S staining and COX-2 immunofluorescence staining for histological analysis.

Supplementary Figures

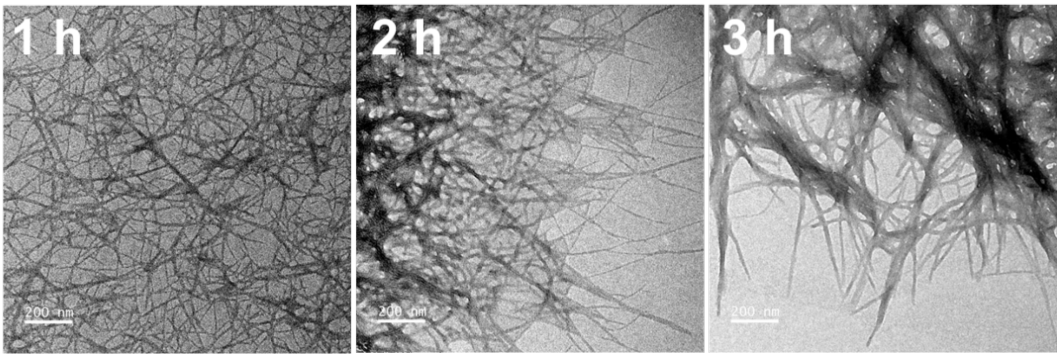

Figure S1. Typical TEM images of Ca-DEX nanoparticles prepared at different stirring time.

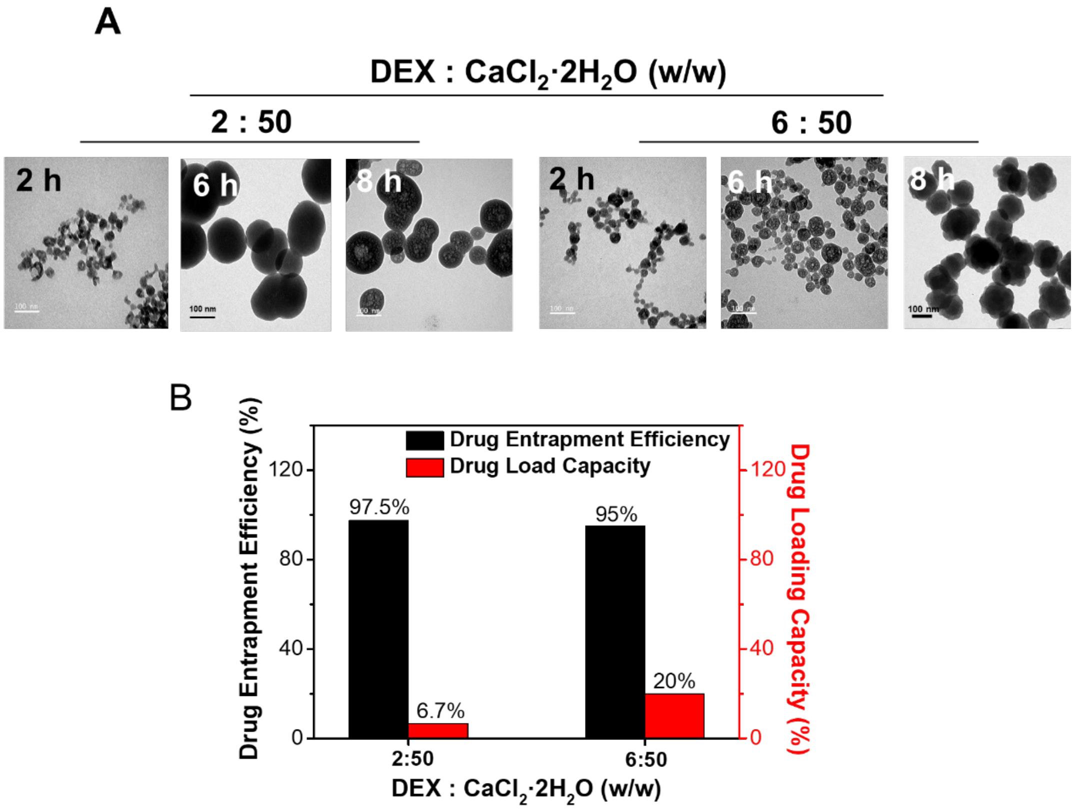

Figure S2. (A) Typical TEM images of $CaCO_3$-DEX nanoparticles prepared at different feeding ratios of DEX and $CaCl_2 \cdot 2H_2O$ and different reaction time. (B) Quantification of DEX-loading capacity (red) and DEX-entrapment efficiency (black) of $CaCO_3$-DEX at different feeding ratios of DEX and $CaCl_2 \cdot 2H_2O$.

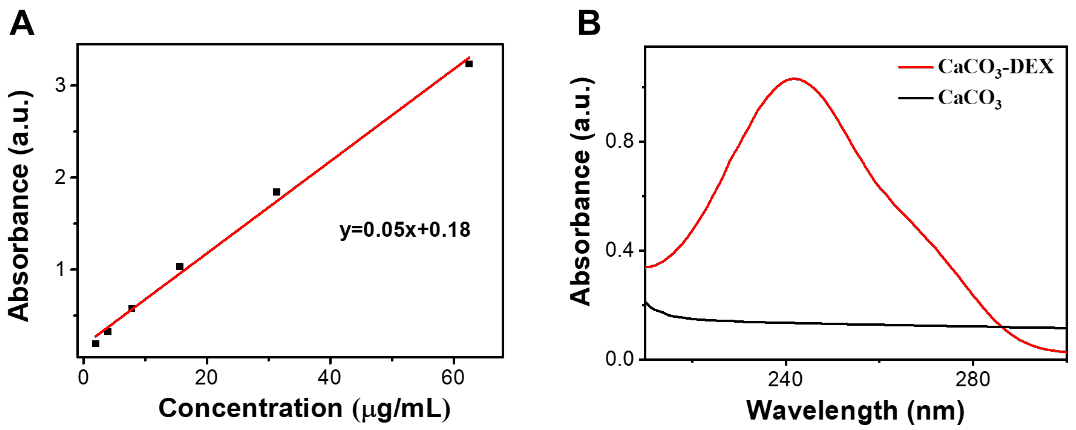

Figure S3. (A) The standard curve of DEX at 241 nm. (B) The absorbance of $CaCO_3$-DEX and $CaCO_3$-DEX-PAH.

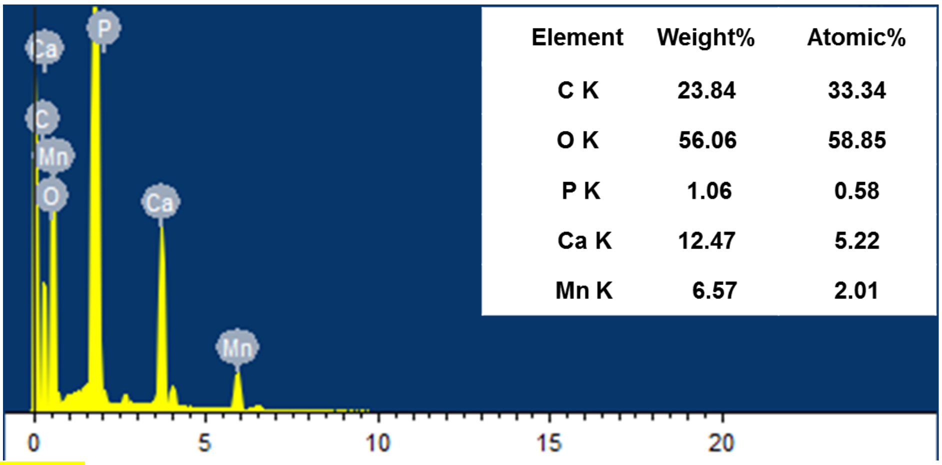

Figure S4. Elemental analysis of Mn:$CaCO_3$-DEX by scanning electron microscope (SEM).

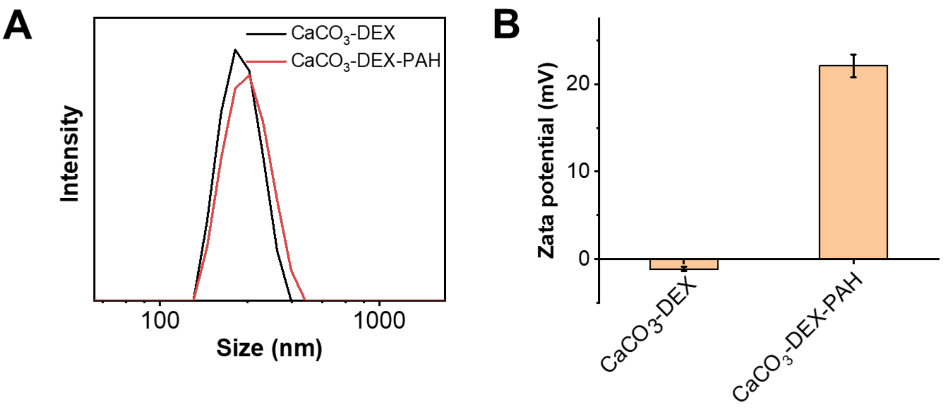

Figure S5. (A) Hydrodynamic diameters and (B) zeta potential of $CaCO_3$-DEX and $CaCO_3$-DEX-PAH.

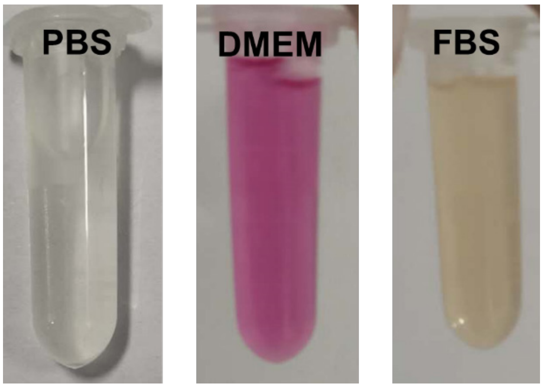

Figure S6. Photographs of Mn:$CaCO_3$-DEX after incubation in PBS (pH 7.4), cell medium (DMEM), and serum (FBS) for 12 h.

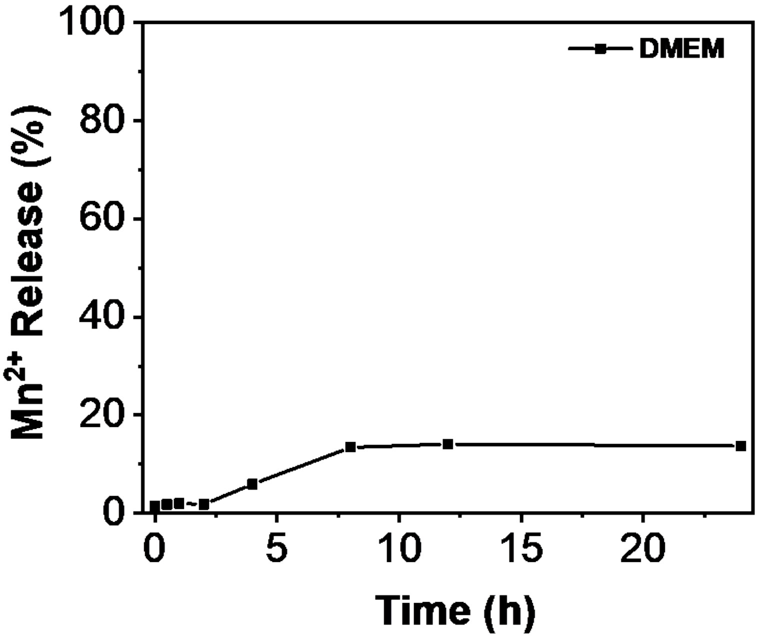

Figure S7. Stability study of Mn:$CaCO_3$-DEX in DMEM for 24 h.

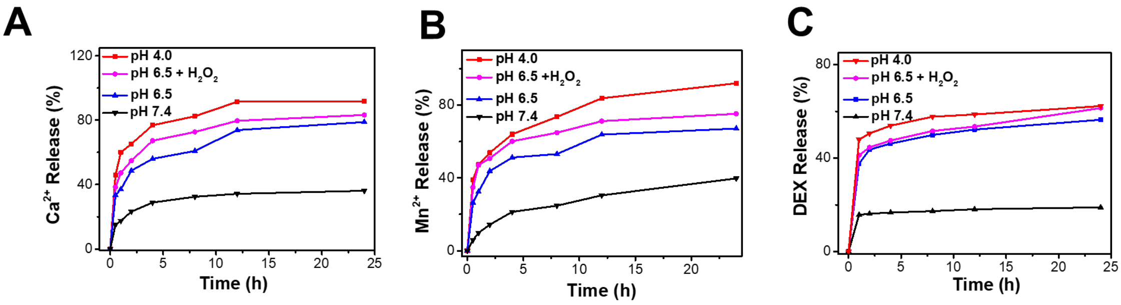

Figure S8. Time-dependent release profile of (A) Ca^{2+}, (B) Mn^{2+} and (C) DEX incubated with different PBS solutions (pH 7.4, pH 6.5, pH 6.5 + H_2O_2 and pH 4.0).

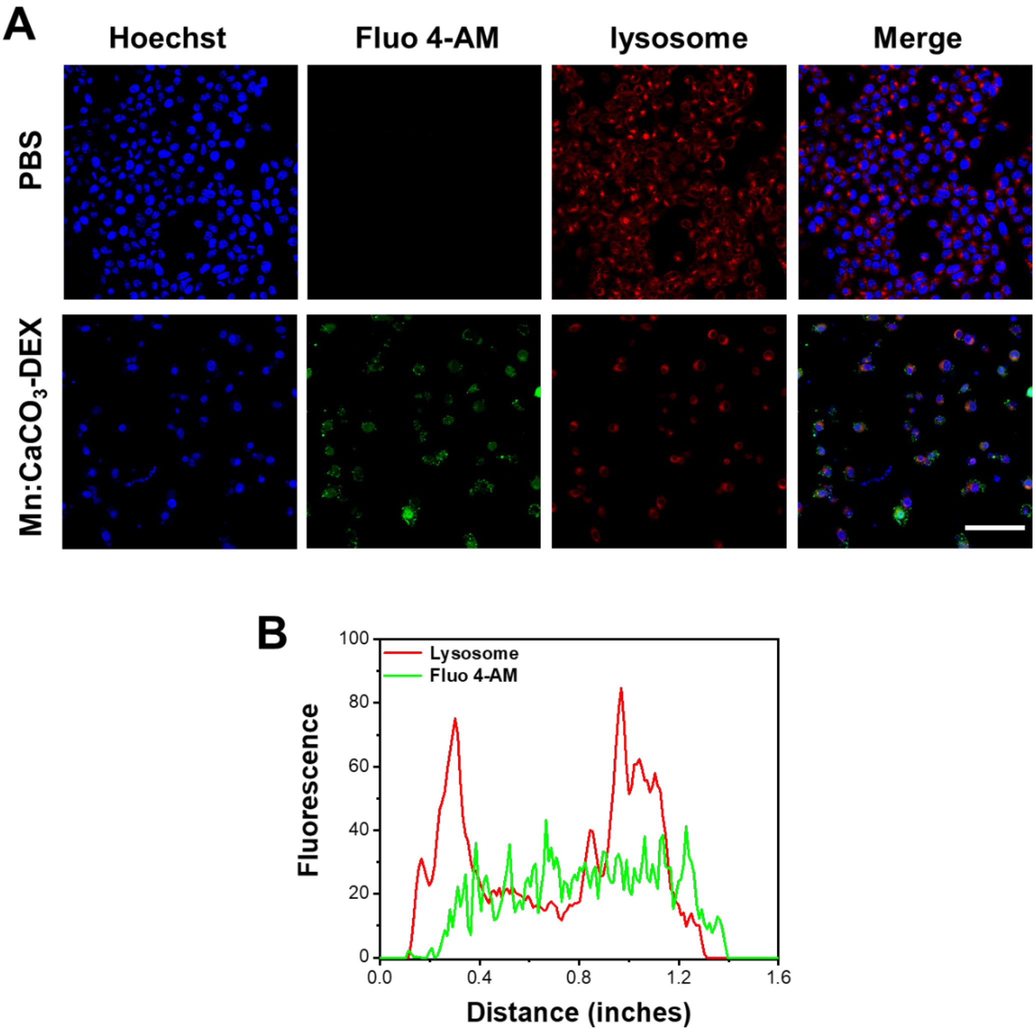

Figure S9. (A) Co-localization of Ca^{2+} (Fluo 4-AM, green), lysosome (red) and nucleus (blue) after incubation with PBS and Mn:$CaCO_3$-DEX . (B) Corresponding fluorescence profiles of Mn:$CaCO_3$-DEX group (scale bar, 50 μm).

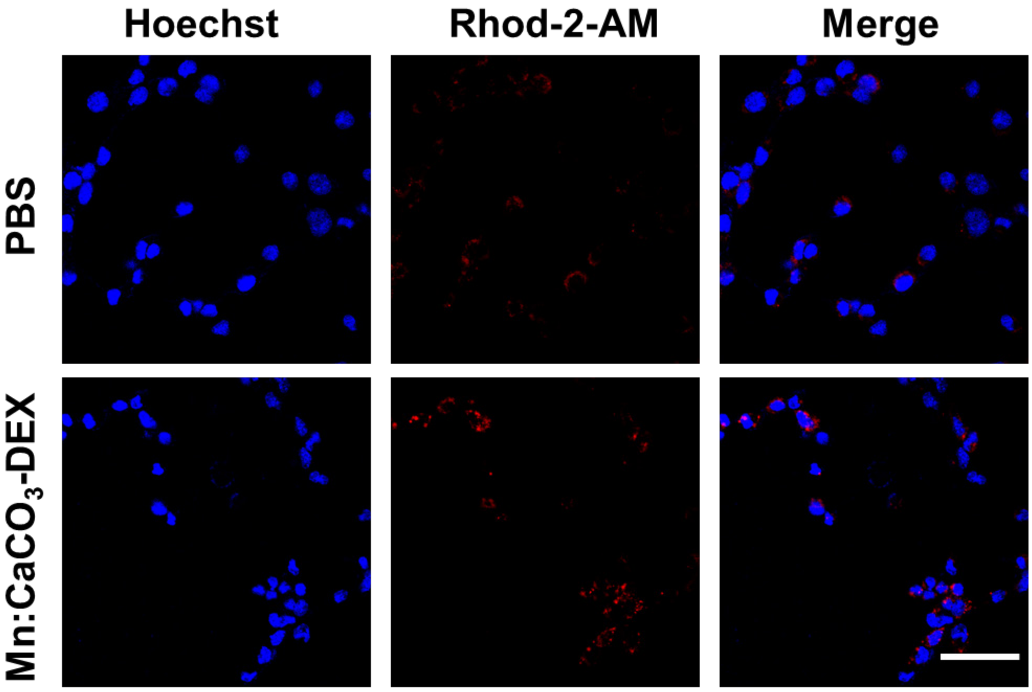

Figure S10. CLSM evaluation of Ca^{2+} accumulate in mitochondria on U87MG cells incubated with Mn:CaCO3-DEX detected by Rhod-2-AM (scale bar, 50 μm).

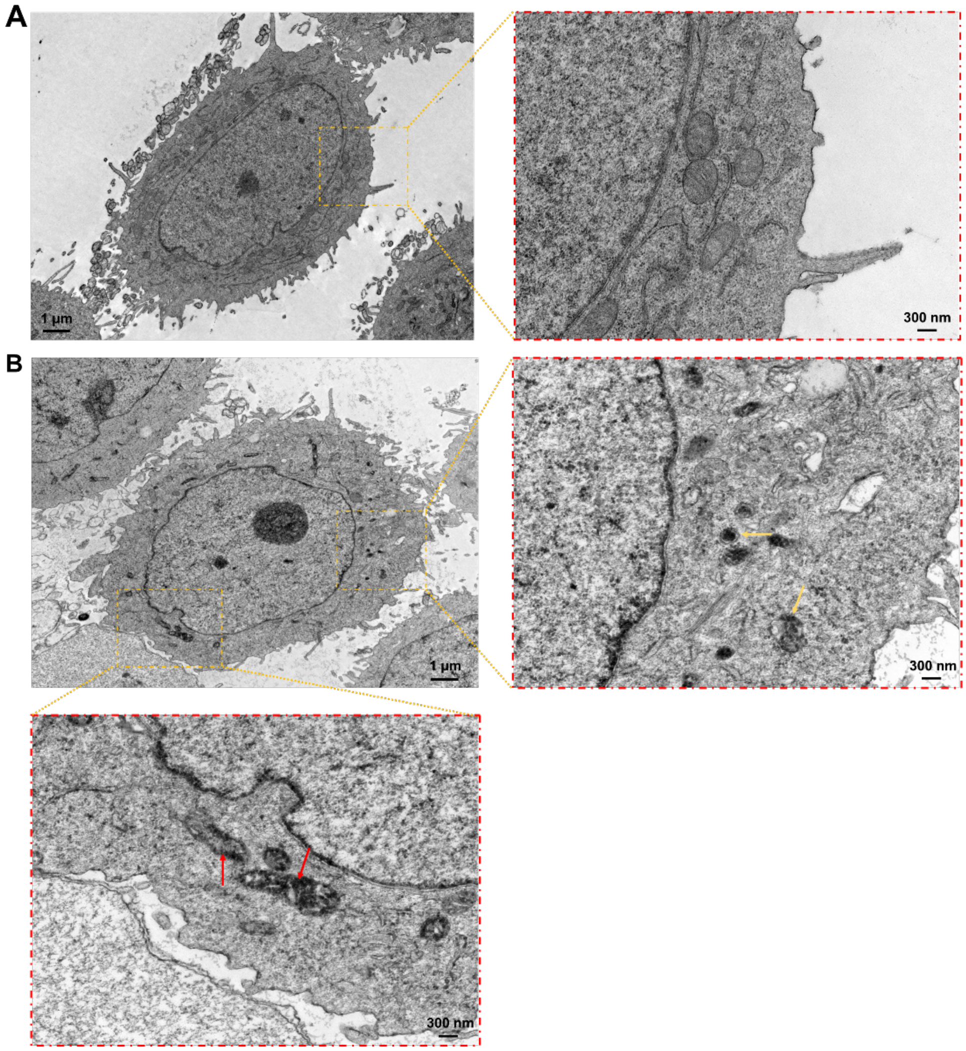

Figure S11. Bio-TEM images of Hep G2 cells after incubation with (A) PBS or (B) Mn:$CaCO_3$-DEX for 24 h.

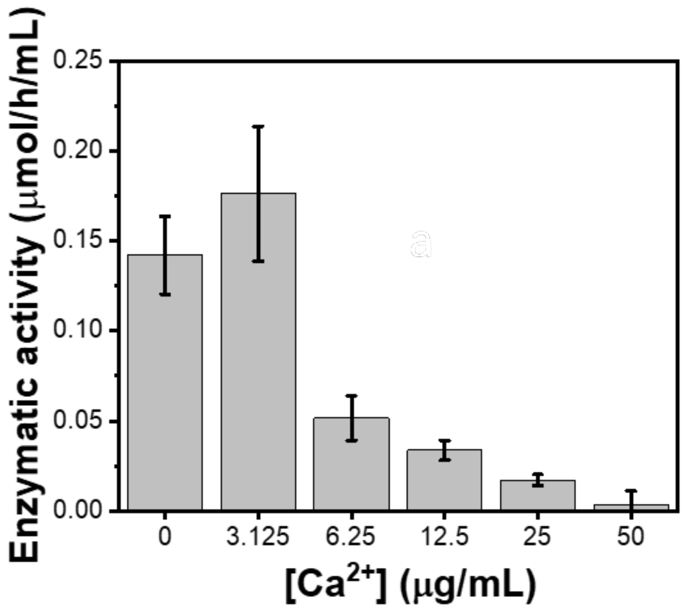

Figure S12. The enzymatic activity of U87MG cells after incubation with different concentration of Mn:$CaCO_3$-DEX.

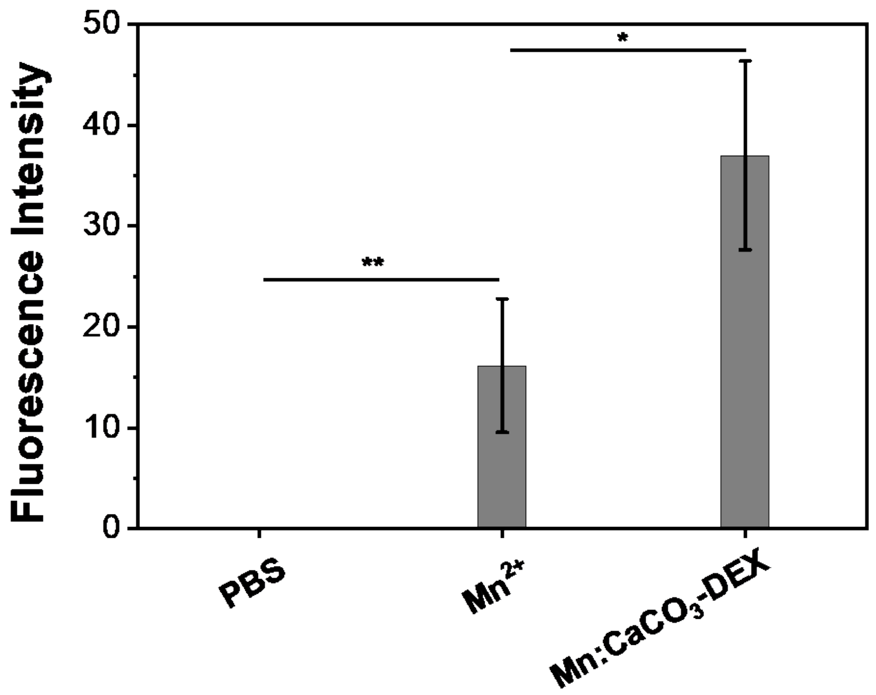

Figure S13. The fluorescence intensity of ·OH, based on DCFH-DA staining results in panel figure 2c (*$P < 0.05$, **$P < 0.01$).

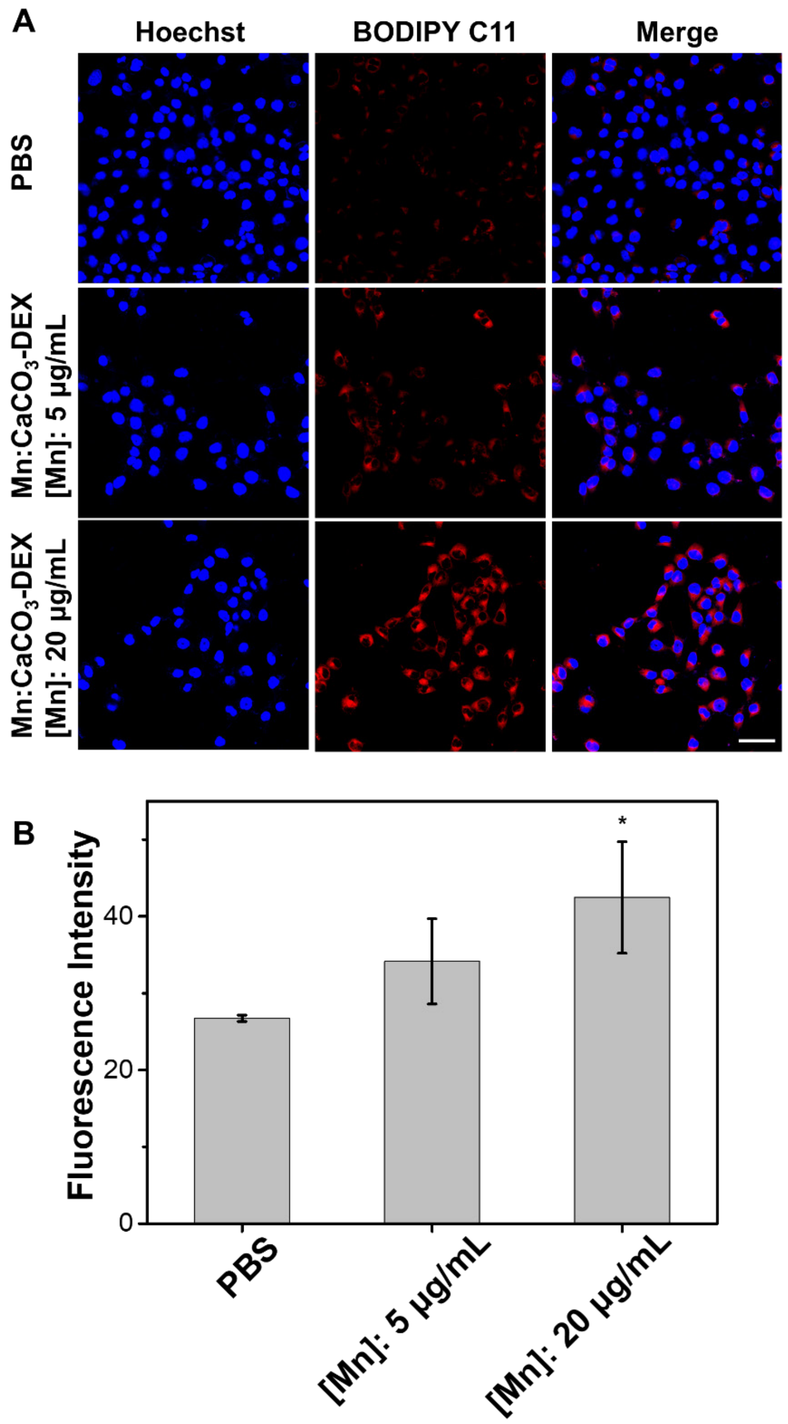

Figure S14. (A) CLSM observation on the intracellular distribution of lipoperoxides in U87MG cells after incubation with PBS and Mn:$CaCO_3$-DEX for 24 hours. The blue and red colors indicate cell nucleus and lipoperoxides, respectively (scale bar, 50 μm). (B) lipoperoxides, based on BODIPY C11 staining results in panel (a) (*$P < 0.05$).

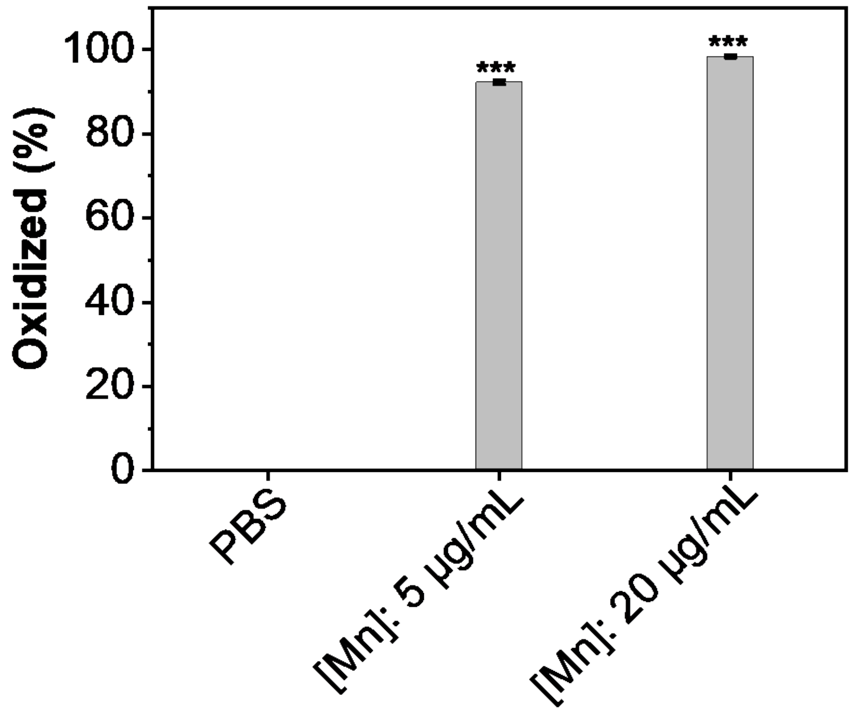

Figure S15. The fraction of oxidized BODIPY C11 (***P < 0.001).

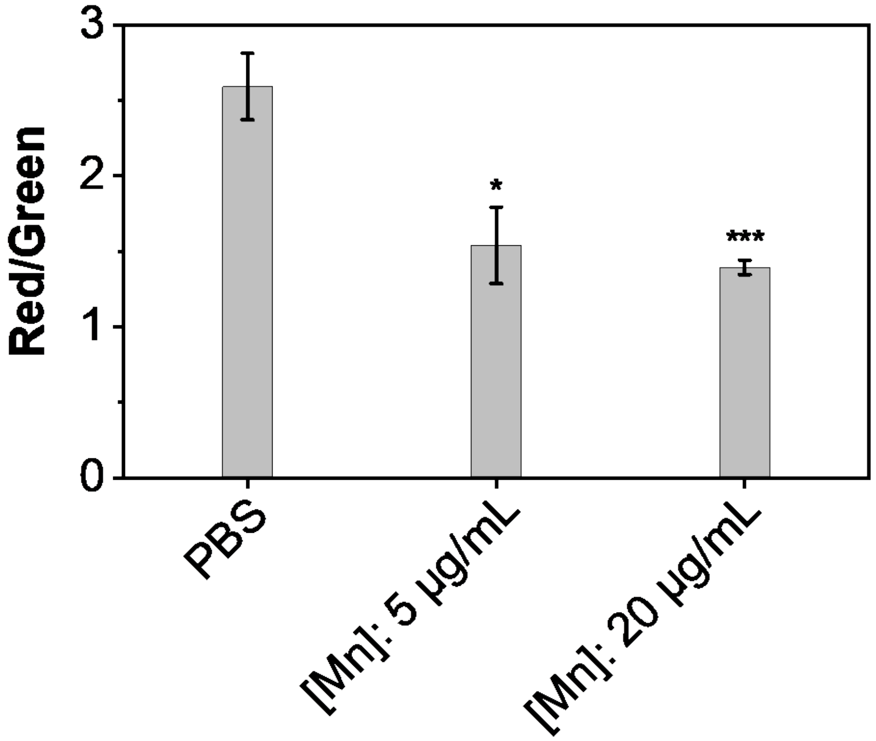

Figure S16. The membrane potential (ΔΨm) changes, assessed by JC-1staining (*P < 0.05; ***P < 0.001).

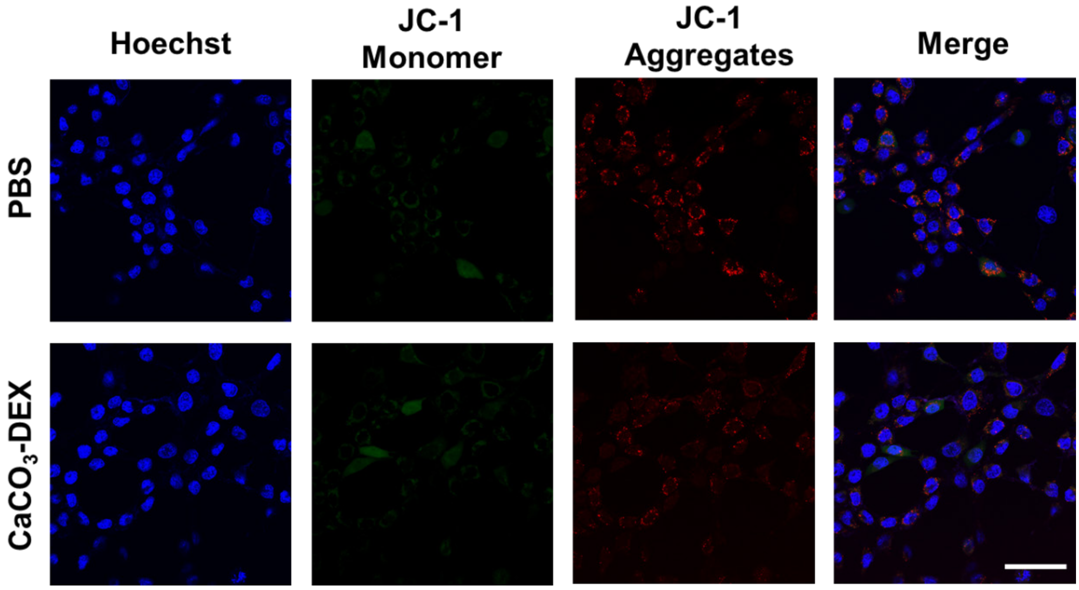

Figure S17. CLSM observation on the changes in the mitochondrial membrane potential of U87MG cells after incubation with $CaCO_3$-DEX. The blue, red, and green colors indicate cell nucleus, and JC-1 J-aggregates and monomer, respectively (scale bar, 50 μm).

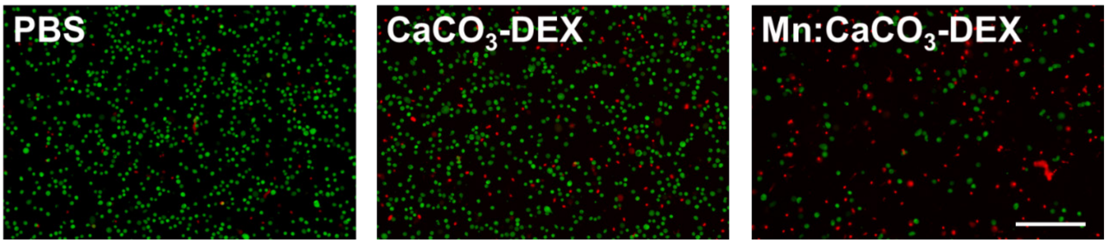

Figure S18. Calcein-AM and PI co-stained U87MG cells with $CaCO_3$-DEX or Mn:$CaCO_3$-DEX (scale bar, 100 μm).

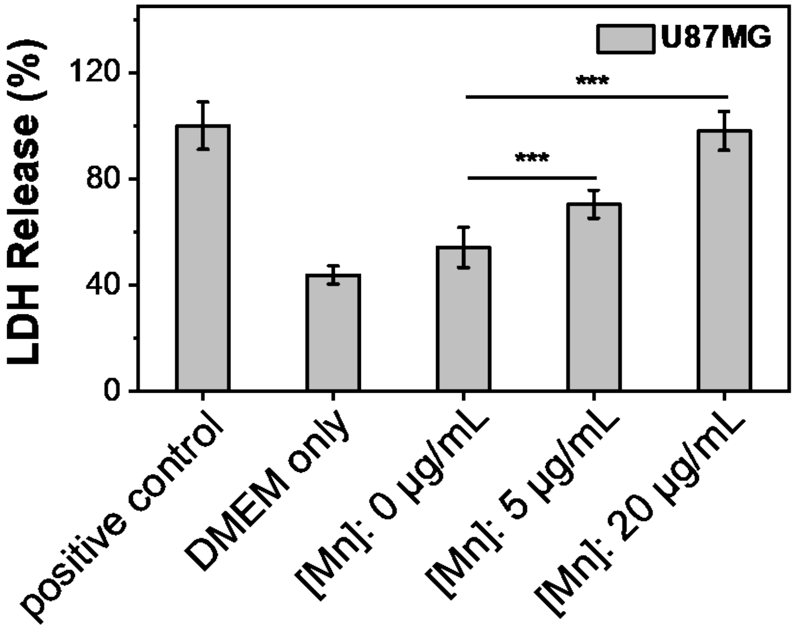

Figure S19. LDH release assay after incubation with different concentration of Mn:$CaCO_3$-DEX for U87MG cells (***P < 0.001).

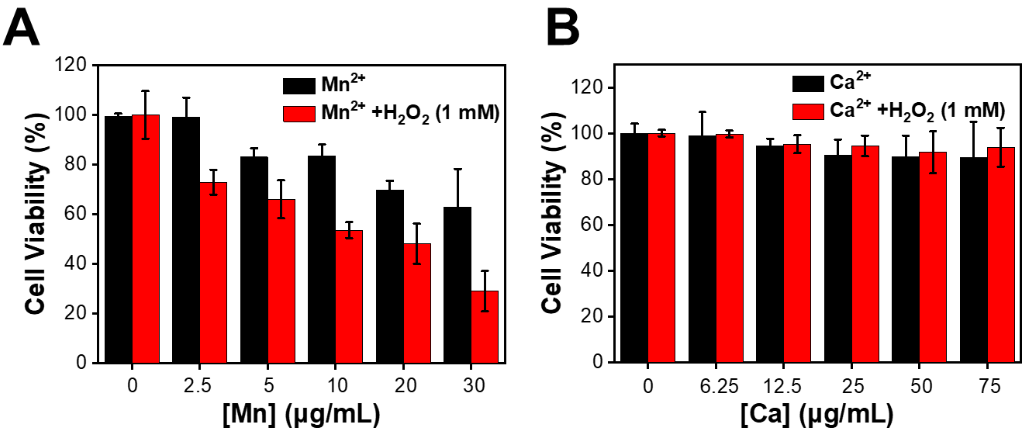

Figure S20. Cell viabilities of Hep G2 cells incubated with different concentrations of (A) Mn^{2+} with and without H_2O_2, and (B) Ca^{2+} with and without the H_2O_2.

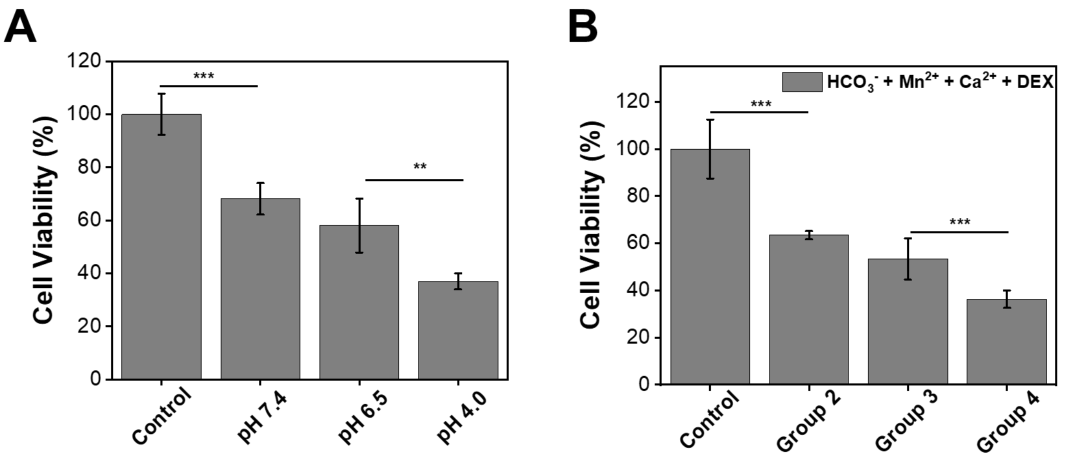

Figure S21. (A) Cell viabilities of Hep G2 cells incubated with Mn:$CaCO_3$-DEX ([Mn]: 10 μg/mL; [Ca]: 25 μg/mL; [DEX]: 20 μg/mL) in different buffer solutions (pH 7.4, 6.5 and 4.0). (B) Cell viabilities of Hep G2 cells incubated with different concentrations of Mn^{2+}, Ca^{2+}, DEX, and HCO_3^- (Maximum release of Mn:$CaCO_3$-DEX ([Mn]: 10 μg/mL; [Ca]: 25 μg/mL; [DEX]: 20 μg/mL). Group 1: Maximum release in pH 7.4 ([Mn]: 4.0 μg/mL; [Ca]: 9.0 μg/mL; [DEX]: 3.8 μg/mL; [HCO_3^-]: 13.8 μg/mL); Group 2: Maximum release in pH 6.5 ([Mn]: 6.7 μg/mL; [Ca]: 19.7 μg/mL; [DEX]: 11.3 μg/mL); Group 3: Maximum release in pH 4.0; ([Mn]: 9.2 μg/mL; [Ca]: 23.0 μg/mL; [DEX]: 12.5 μg/mL). (**$P < 0.01$; ***$P < 0.001$)

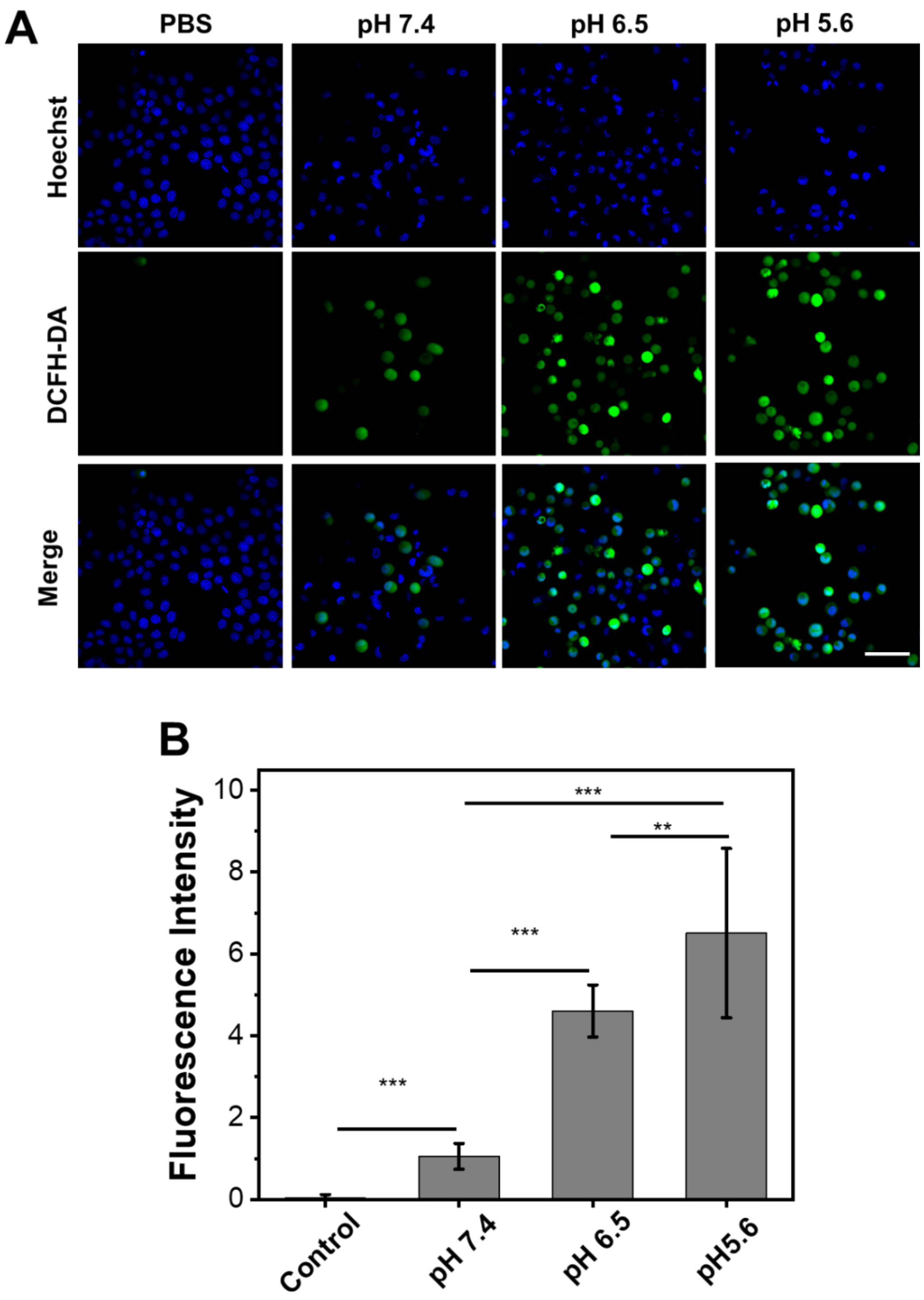

Figure S22. (A) Intracellular ·OH generation after incubation with Mn:$CaCO_3$-DEX in different buffer solutions (pH 7.4, 6.5, and 5.6) detected by DCFH-DA probe. The blue and green fluorescence indicate cell nucleus and DCFH-DA, respectively (scale bar, 50 μm). (B) The fluorescence intensity of ·OH ,based on DCFH-DA staining resuts in panel (a) (**$P < 0.01$; ***$P < 0.001$).

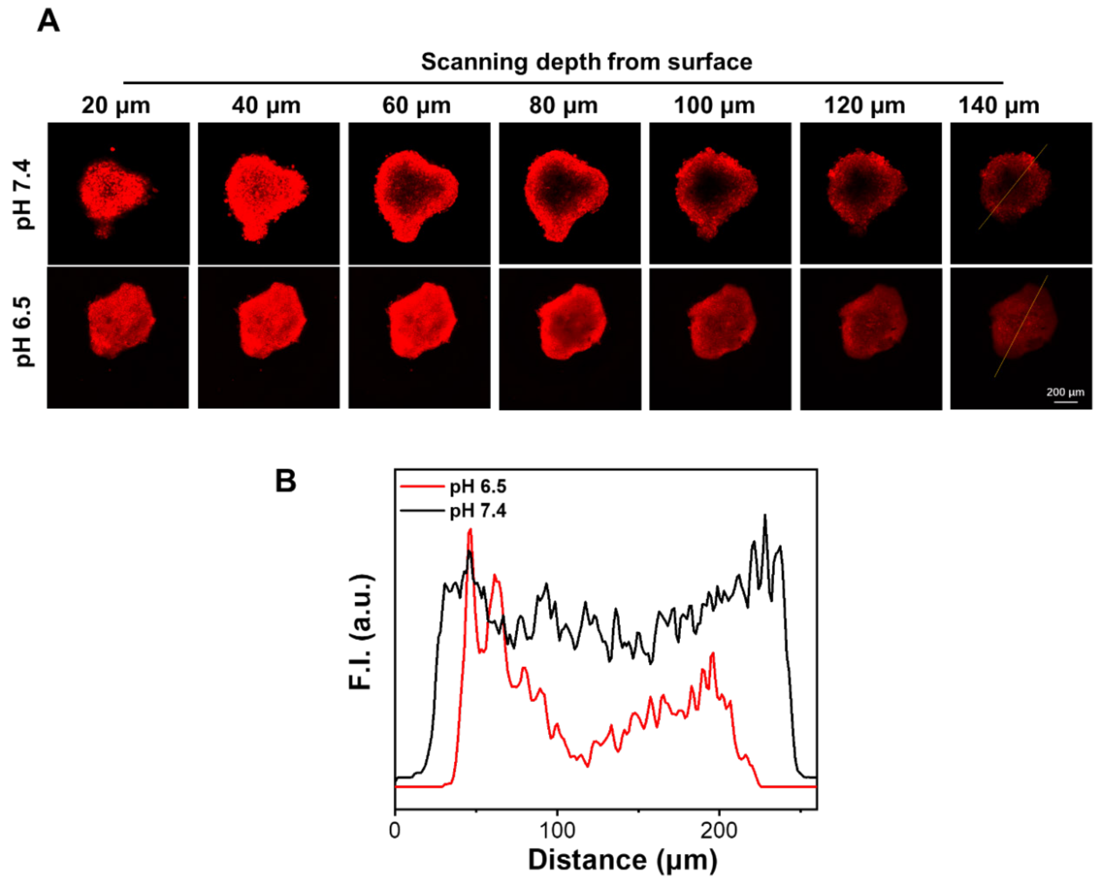

Figure S23. (A) In vitro penetration into Hep G2 multicellular tumor spheroids (MTSs) after treated with Mn:$CaCO_3$-DEX-Cy5.5 in different media (pH 6.5 or 7.4) for 24 h, and Z-stack pictures were taken from top to equatorial plane of MTSs in 20 μm thickness (Scale bar, 200 μm). (B) Corresponding fluorescence profiles in (a).

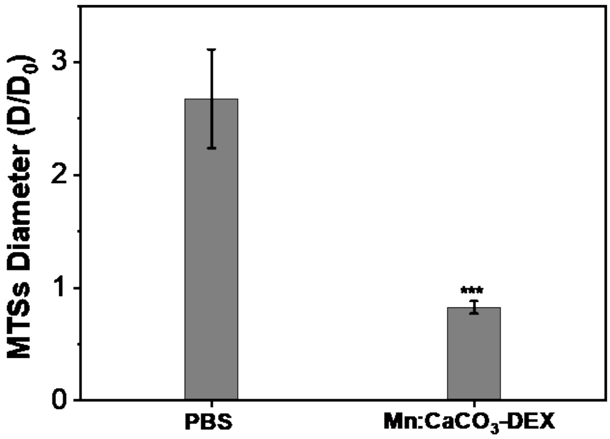

Figure S24. Growth inhibitory effect of the Mn:$CaCO_3$-DEX ([Mn]: 50 μg/mL) for 3D MTSs in acidic buffer solutions (pH 6.5) (***$P < 0.001$).

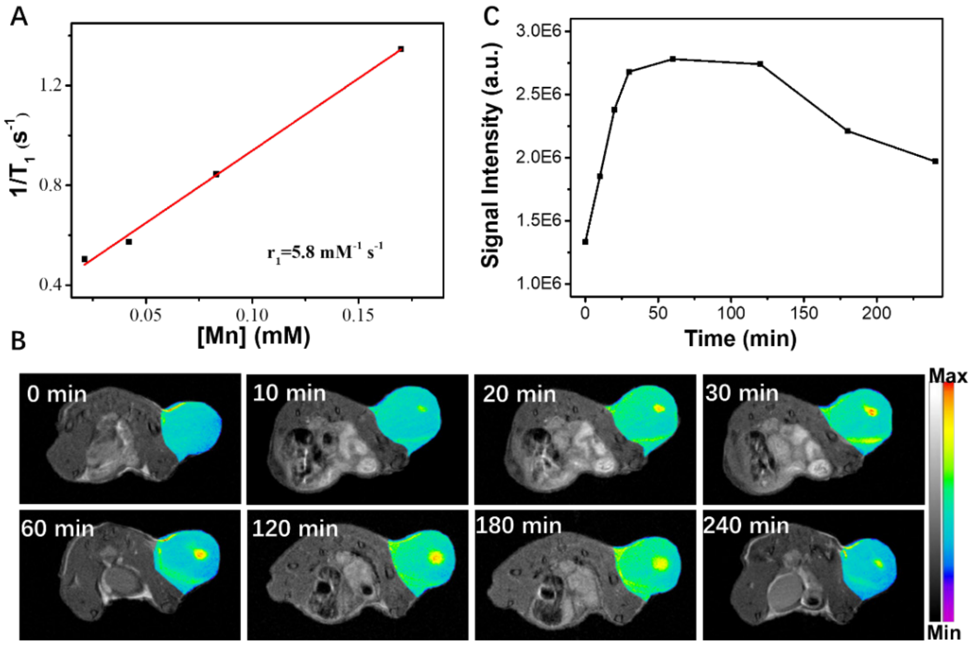

Figure S25. (A) The relaxation rate (r_1) value of Mn:$CaCO_3$-DEX after incubated under acidic environment (pH 6.5) in the presence of H_2O_2. (B) T_1-MRI images of BALB/C nude subcutaneous tumor-bearing mice before and after local injection of Mn:$CaCO_3$-DEX within tumor area. (C) T_1-MRI signal of tumor area strength before and after injection of Mn:$CaCO_3$-DEX.

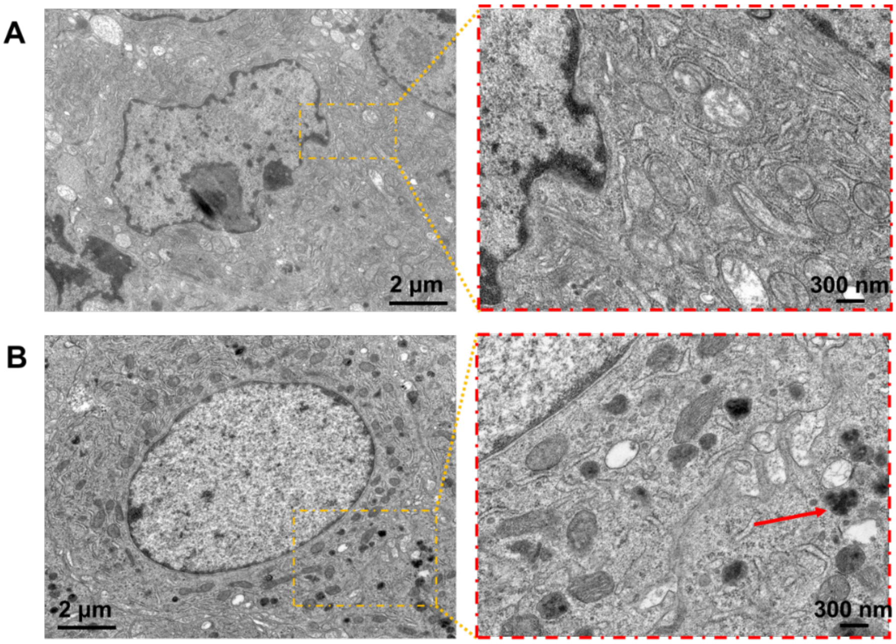

Figure S26. Bio-TEM images of tumor tissues after treatment with (A) PBS and (B) Mn:$CaCO_3$-DEX ([Mn]: 2 mg/kg, [DEX]: 4 mg/kg, [Ca]: 5 mg/kg).

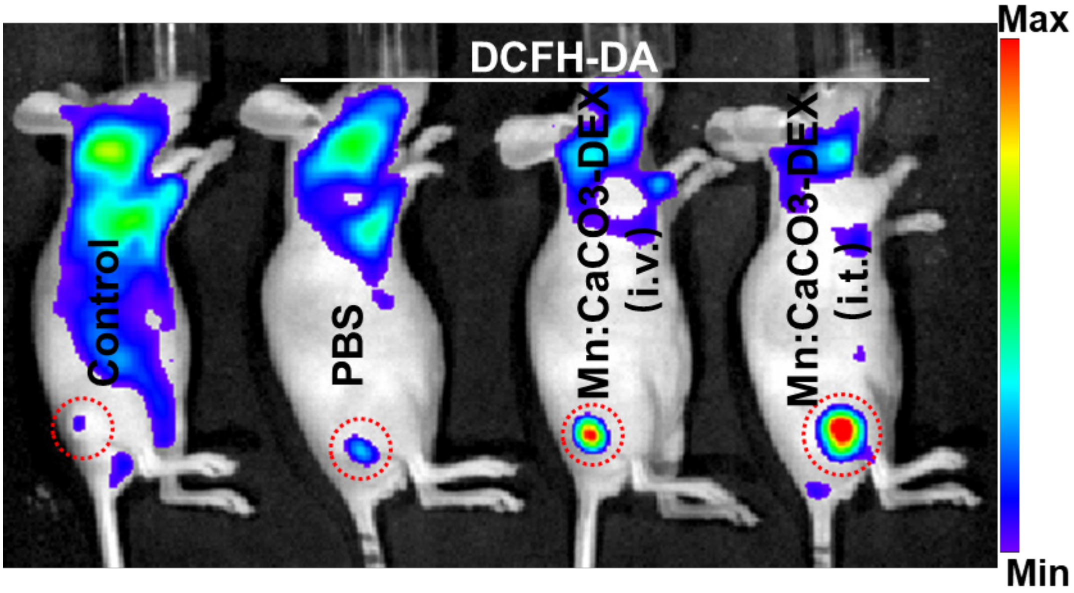

Figure S27. The generation of ·OH in tumor after different treatment detected by DCFH-DA probe. Control: without DCFH-DA; PBS: PBS (i.v.) with DCFH-DA (i.t.); Mn:$CaCO_3$-DEX (i.v.) injection: Mn:$CaCO_3$-DEX (i.v.) with DCFH-DA,and Mn:$CaCO_3$-DEX (i.t.): Mn:$CaCO_3$-DEX (i.t.) with DCFH-DA

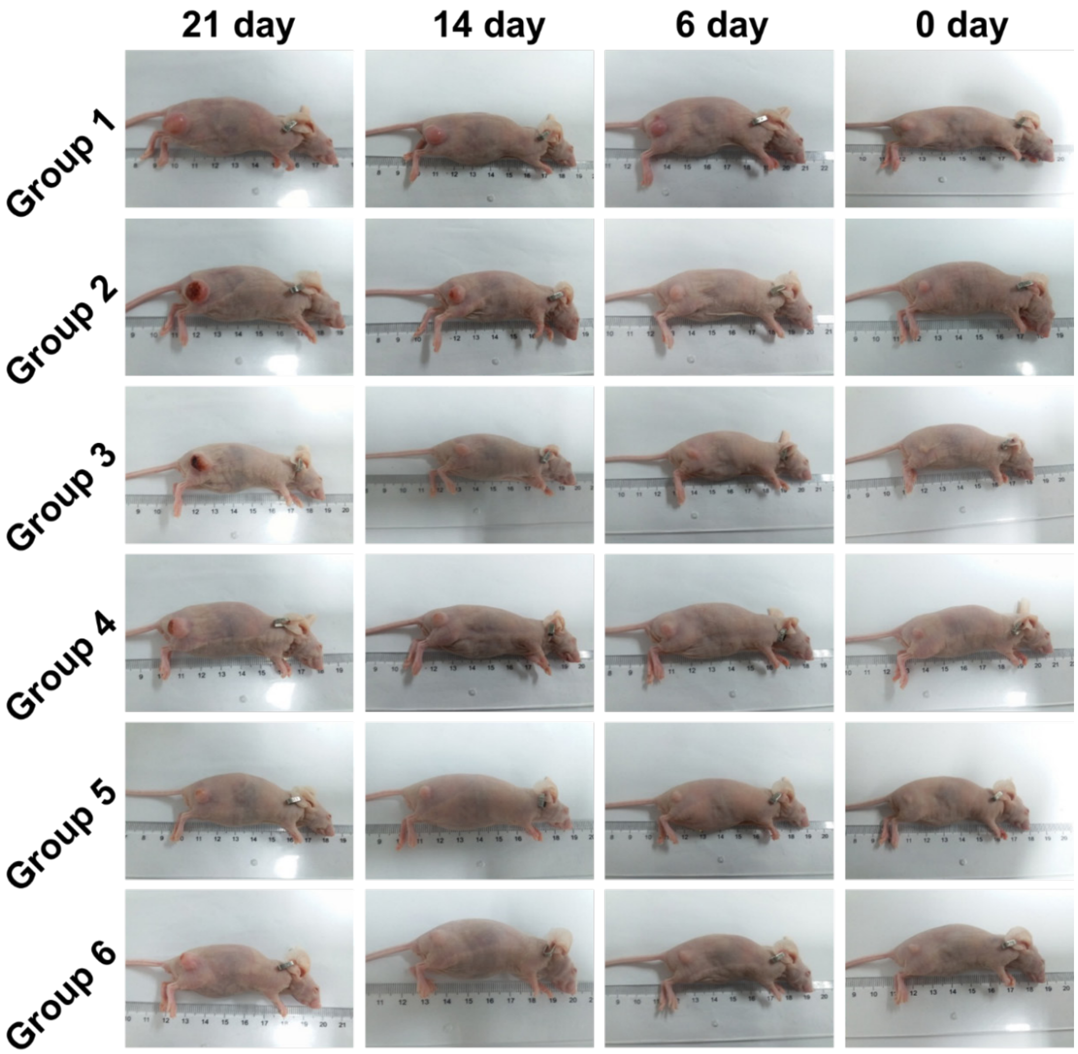

Figure S28. Body photographs of BALB/C nude subcutaneous tumor-bearing mice in different formulations during the 21-day treatment period. Groups 1, 2, 3, 4, 5 and 6 were used to represent PBS; DEX (4 mg/kg, one dose); $CaCO_3$-DEX ([DEX]: 4 mg/kg, [Ca]: 5 mg/kg, one dose); 2×Mn:$CaCO_3$-DEX ([Mn]: 4 mg/kg, [DEX]: 8 mg/kg, [Ca]: 10 mg/kg, one dose); Mn:$CaCO_3$-DEX ([Mn]: 2 mg/kg, [DEX]: 4 mg/kg, [Ca]: 5 mg/kg, four doses); 2×Mn:$CaCO_3$-DEX ([Mn]: 4 mg/kg, [DEX]: 8 mg/kg, [Ca]: 10 mg/kg, four doses).

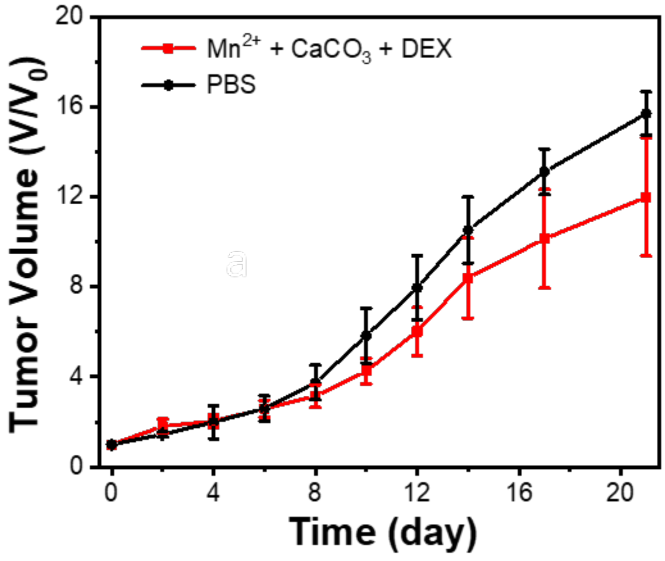

Figure S29. Tumor growth curves of U87MG tumor-bearing mice exposed to different formulations (PBS and $MnCl_2$+ $CaCO_3$ +DEX ([Mn]: 4 mg/kg, [DEX]: 8 mg/kg, [Ca]: 10 mg/kg, four doses) after the 21-day treatment period.

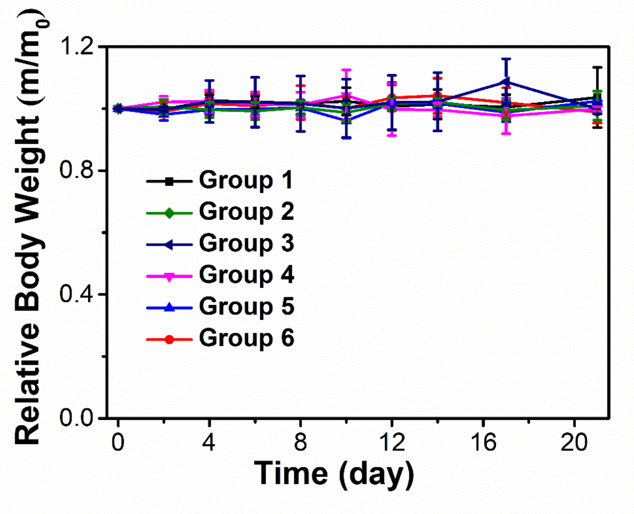

Figure S30. Body weight curves of BALB/C nude subcutaneous tumor-bearing mice in different formulations during the 21-day treatment period. Groups 1, 2, 3, 4, 5 and 6 were used to represent PBS; DEX (4 mg/kg, one dose); $CaCO_3$-DEX ([DEX]: 4 mg/kg, [Ca]: 5 mg/kg, one dose); 2×Mn:$CaCO_3$-DEX ([Mn]: 4 mg/kg, [DEX]: 8 mg/kg, [Ca]: 10 mg/kg, one dose); Mn:$CaCO_3$-DEX ([Mn]: 2 mg/kg, [DEX]: 4 mg/kg, [Ca]: 5 mg/kg, four doses); 2×Mn:$CaCO_3$-DEX ([Mn]: 4 mg/kg, [DEX]: 8 mg/kg, [Ca]: 10 mg/kg, four doses).

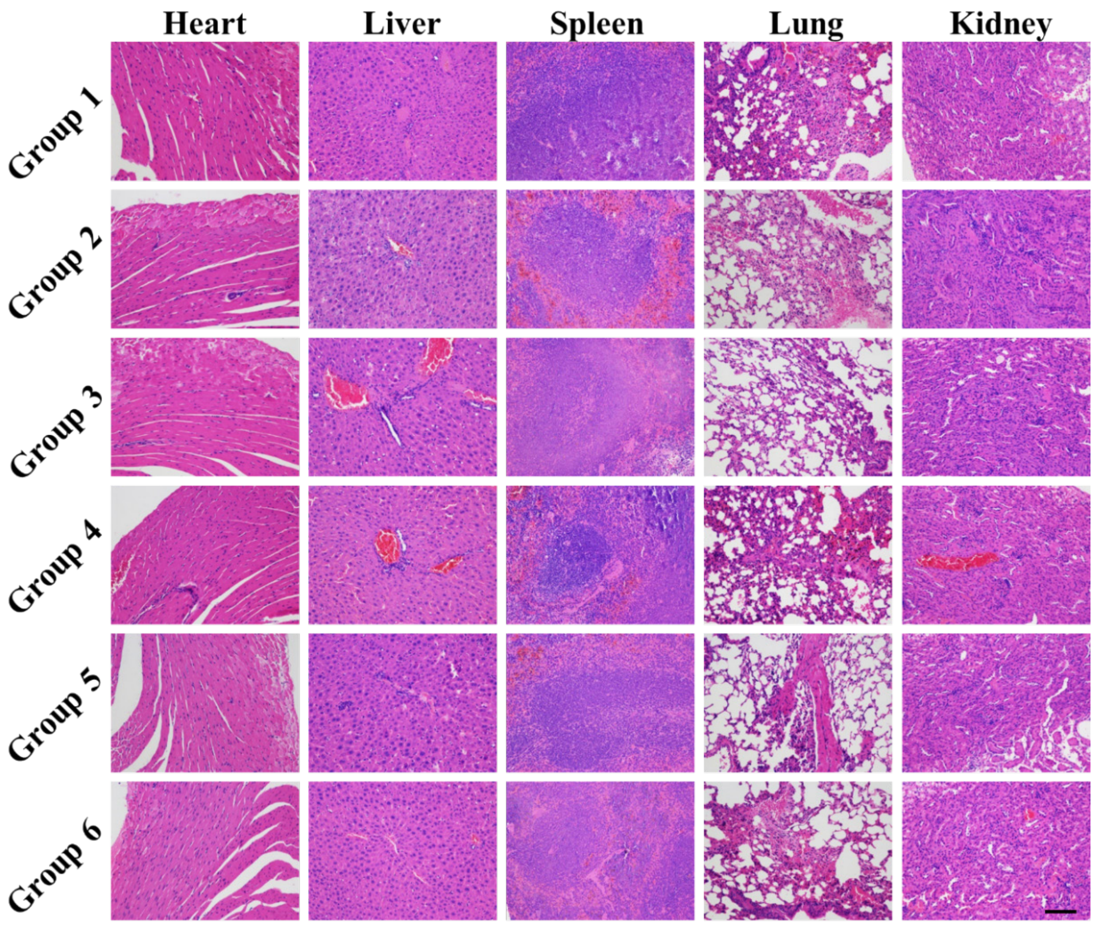

Figure S31. Hematoxylin & eosin (H&E)-stained of the organ harvested from BALB/C nude subcutaneous tumor-bearing mice in different formulations after the 21-day treatment period (scale bar:100 μm). Groups 1, 2, 3, 4, 5 and 6 were used to represent PBS; DEX (4 mg/kg, one dose); $CaCO_3$-DEX ([DEX]: 4 mg/kg, [Ca]: 5 mg/kg, one dose); 2×Mn:$CaCO_3$-DEX ([Mn]: 4 mg/kg, [DEX]: 8 mg/kg, [Ca]: 10 mg/kg, one dose); Mn:$CaCO_3$-DEX ([Mn]: 2 mg/kg, [DEX]: 4 mg/kg, [Ca]: 5 mg/kg, four doses); 2×Mn:$CaCO_3$-DEX ([Mn]: 4 mg/kg, [DEX]: 8 mg/kg, [Ca]: 10 mg/kg, four doses).

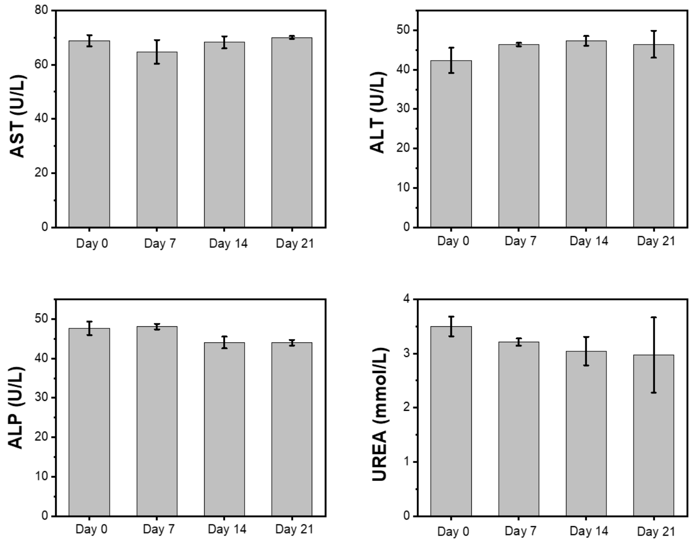

Figure S32. Serum chemistry of mice treated with Mn:$CaCO_3$-DEX ([Mn]: 4 mg/kg, [DEX]: 8 mg/kg, [Ca]: 10 mg/kg). Blood samples were collected on day 0, day 7, 14 and day 21. AST, aspartate transaminase; ALT, alanine transaminase; ALP, Alkaline Phosphatase; UREA, blood urea nitrogen.

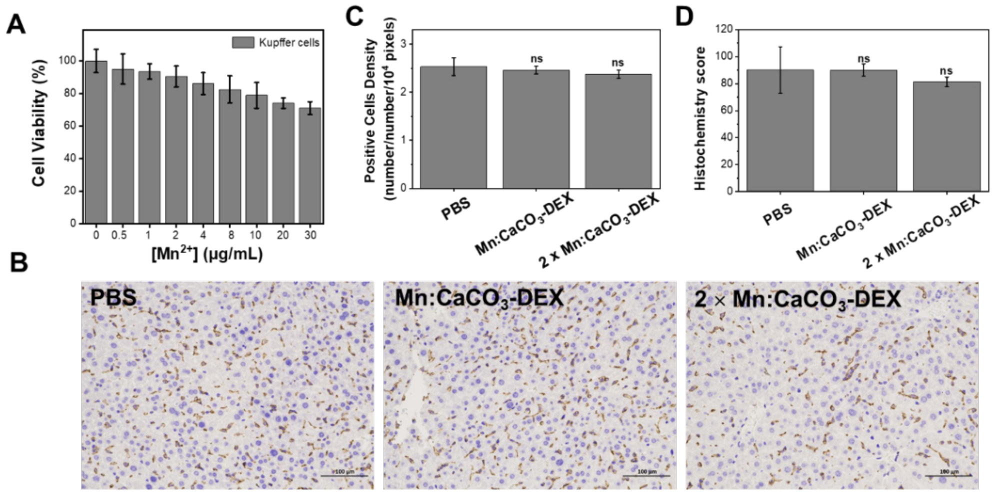

Figure S33. (A) Cell viability of Kupffer cells incubated with different concentrations of Mn:$CaCO_3$-DEX. (B) Liver sections were immunostained with anti-F4/80 antibody, nucleus was stained with Hoechst. (C,D) Statistical results of (C) the number of positive cells density per field and (D) histochemistry score, based on F4/80 staining results in panel (B) (ns: not significant). Mn:$CaCO_3$-DEX ([Mn]: 2 mg/kg, [DEX]: 4 mg/kg, [Ca]: 5 mg/kg, four doses); 2×Mn:$CaCO_3$-DEX ([Mn]: 4 mg/kg, [DEX]: 8 mg/kg, [Ca]: 10 mg/kg, four doses).

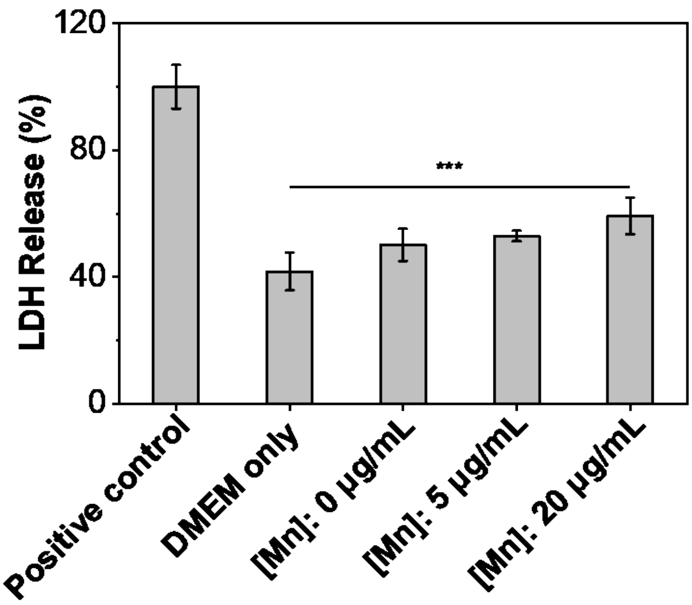

Figure S34. LDH release assay after incubated with different concentration of Mn:$CaCO_3$-DEX for Hep G2 cells (***P < 0.001).

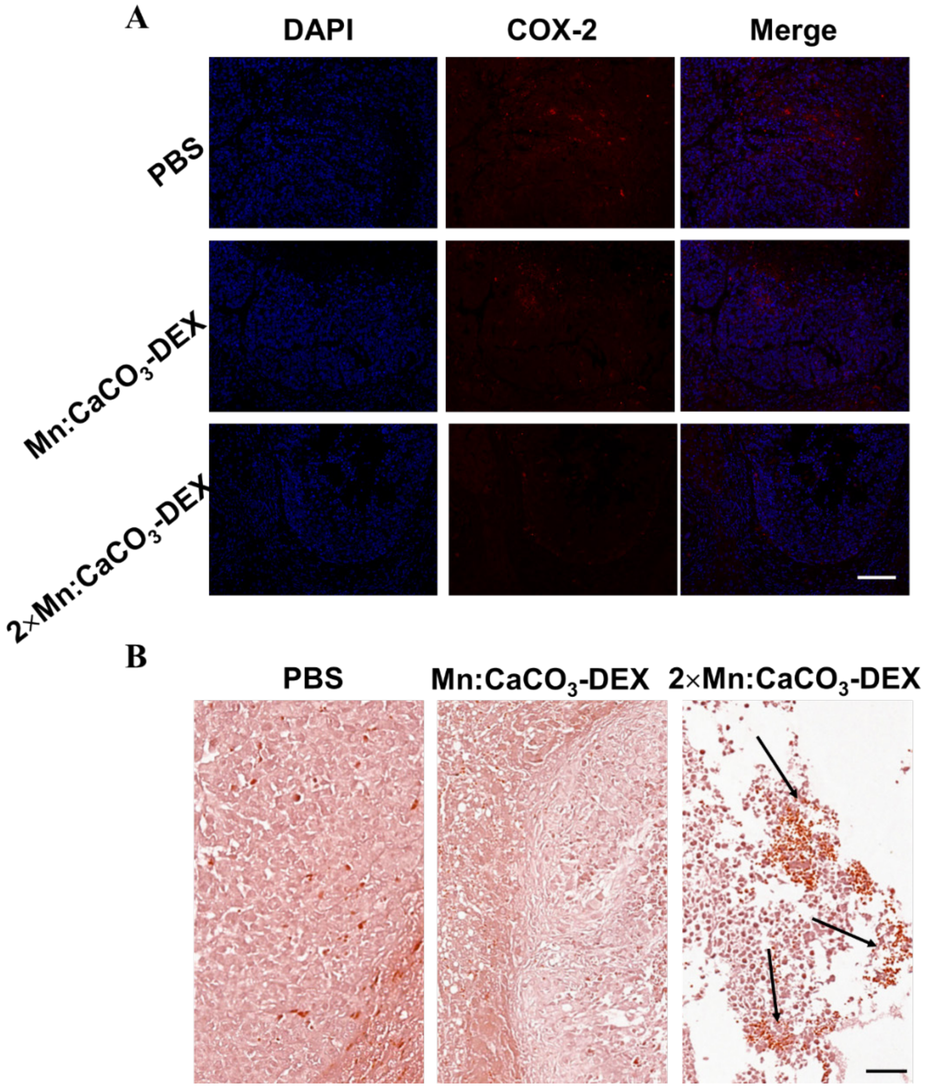

Figure S35. the staining results of (A) COX-2 immunofluorescence (scale bar:200 μm) and (B) Alizarin Red S (scale bar:100 μm) of BALB/C nude orthotopic hepatic tumors-bearing mice after different formulations. Mn:$CaCO_3$-DEX ([Mn]: 2 mg/kg, [DEX]: 4 mg/kg, [Ca]: 5 mg/kg, four doses); 2×Mn:$CaCO_3$-DEX ([Mn]: 4 mg/kg, [DEX]: 8 mg/kg, [Ca]: 10 mg/kg, four doses).

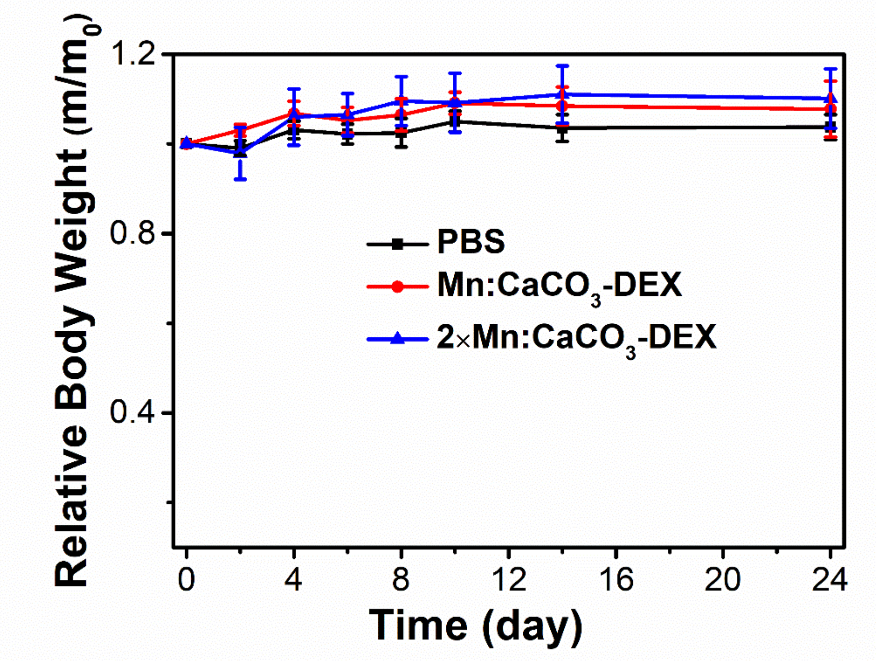

Figure S36. Body weight curves of BALB/C nude orthotopic hepatic tumors-bearing mice after different formulations during the 24-day treatment period (n = 5). Mn:$CaCO_3$-DEX ([Mn]: 2 mg/kg, [DEX]: 4 mg/kg, [Ca]: 5 mg/kg, four doses); 2×Mn:$CaCO_3$-DEX ([Mn]: 4 mg/kg, [DEX]: 8 mg/kg, [Ca]: 10 mg/kg, four doses).
